# CDC50A might be a novel biomarker of epithelial ovarian cancer-initiating cells

**DOI:** 10.1186/s12885-022-09953-y

**Published:** 2022-08-19

**Authors:** Jie Yin, Yiping Wen, Jing Zeng, Yanyan Zhang, Jiayu Chen, Yanmei Zhang, Tiantian Han, Xiaoying Li, Hong Huang, Yan Cai, Ying Jin, Yan Li, Wei Guo, Lingya Pan

**Affiliations:** 1 Department of Obstetrics and Gynecology, Peking Union Medical College Hospital, Chinese Academy of Medical Science and Peking Union Medical College, 1 Shuai Fu Yuan, Dongcheng district, Beijing, 100730 China; 2 Department of Obstetrics and Gynecology, Union Hospital, Tongji Medical College, Huazhong University of Science and Technology, Wuhan, Hubei China; 3 Department of Basic Medicine, Center for Stem Cell Biology and Regenerative Medicine, School of Medicine, Tsinghua University, Beijing, China

**Keywords:** CDC50A, Cancer stem cell, Epithelial ovarian cancer, Prognosis

## Abstract

**Background:**

The aim of this work was to screen and validate biomarkers of ovarian cancer-initiating cells to detect the mechanisms of recurrence of epithelial ovarian cancer (EOC).

**Methods:**

Stably labelled the amino acid in side population (SP) cells of epithelial ovarian cancer which were rich in cancer-initiating cells and non-SP cells with isotope in culture and differentially expressed cellular membrane proteins in SP cells were identified through proteomics technology. The new candidate biomarker was screened and validated through RT-PCR and western blot. Both in cell lines and primary EOC, cancer-initiating biofunctions of CDC50A positive cells were validated. Moreover, the characteristics of mesenchymal transition (EMT) was also detected and the correlation between the biomarker and clinical prognosis was observed.

**Results:**

Through proteomics technology, candidate protein CDC50A was screened, and its significantly differential expression in SP cells was validated. CDC50A-positive cells from cell lines and primary ovarian cancer tissues were validated to show characteristics of cancer-initiating cells both in vitro and in vivo, including sphere-forming, self-renewal, differentiation, tumor metastasis and tumorigenicity in mice. The relationship between CDC50A-positive cells from primary tissues and tumour metastasis was confirmed based on their mesenchymal transition characteristics. Among 16 high-grade ovarian serous cancer patients, a high ratio of CDC50A-positive cells in primary tumours was correlated with a shorter platinum-free interval (*p* = 0.031, HR 0.260, 95% CI 0.77 ~ 0.885).

**Conclusion:**

CDC50A could be used to screen ovarian cancer-initiating cells and might be a new target to resolve tumour development in EOC patients.

**Supplementary Information:**

The online version contains supplementary material available at 10.1186/s12885-022-09953-y.

## Background

Ovarian cancer is the leading cause of gynaecological cancer death [1]. Nearly 70% ~ 80% of patients are diagnosed at an advanced stage because of nonspecific symptoms. Epithelial ovarian cancer (EOC) is the main histopathological type and can be characterized into various subtypes, such as clear cell, endometrioid, mucinous, low-grade serous and high-grade serous. Advanced ovarian cancer is a highly metastatic tumour that spreads throughout the peritoneal cavity. The aim of either primary debulking surgery or interval debulking surgery was to achieve complete removal of macroscopic tumours. Most advanced ovarian cancer patients responded well to platinum-based combination chemotherapy after surgery. The platinum-free interval (PFI) was used to evaluate the patients’ secondary response to chemotherapy. Patients with less than 6 months PFI were defined as having platinum-resistant recurrence [2]. Chemoresistance and frequent recurrence were the main obstacles to improving prognosis.

Understanding the mechanisms of recurrence and drug resistance is essential for improving the prognosis of advanced ovarian cancer patients. In the past 20 years, through proteomics technology, we have screened and validated several biomarkers of platinum resistance in vivo and in vitro, such as annexin A3 [3] and coffilin 1 [4]. However, not all drug resistance occurs in the clinic based on the expression of several proteins because of heterogenicity. The mechanistic details are still not understood. Advances in cancer stem cells (CSCs) have widened our understanding of chemoresistance in EOC. CSCs are a subpopulation of tumour cells with self-renewal and differentiation properties that can sustain tumour growth and recapitulate heterogeneous tumours [5]. CSCs are also thought to be resistant to chemotherapy [6]. Some research into CSCs and their effect on ovarian cancer progression has aided our understanding of chemoresistance. It has been shown that multiple chemotherapy treatment rounds can enrich the CSC population. CSCs maintain a state of quiescence remaining in G_0_ for a prolonged period of time. This presents issues, as most treatments administer therapeutics that target actively dividing cells in the S or M phases. However, we still know little about the location and surface markers of ovarian cancer stem cells. The identification of CSCs has relied on various cell surface markers, such as CD44 in breast cancer [7] and CD24 and CD133 in colorectal cancer [8]. However, no one ovarian cancer-specific surface biomarker was identified and suitable for isolating ovarian cancer-initiating cells. The lack of specific markers severely hinders further clinical and molecular characterization of CSCs in ovarian cancers.

Goodwell et al. first reported a small population of cells showing a distinct fluorescence-activated cell sorting (FACS) profile off to the side of the main population due to a more efficient Hoechst dye efflux and lower fluorescence intensity signal [9]. Side population (SP) cells in a flow cytometry assay are defined by the ability of the cells to rapidly efflux the Hoechst dye through the membrane-spanning ATP-binding cassette (ABC) family of transporters. SP cells identified through Hoechst 33342 dye coexpressing aldehyde dehydrogenase show increased tumorigenic ability both in vitro and in vivo. SP cells from ascites of ovarian cancer patients express stem cell-related genes [10–12]. Preliminary experiments have shown that ovarian cancer stem cells were enriched in SP cells [13]. SP cells sorted from epithelial ovarian cancer cell lines SKOV3 and A2780 show stemness [14]. In this study, proteomics technology was used to screen differentially expressed proteins in SP cells. Candidate biomarkers of ovarian cancer-initiating cells were selected and validated in vivo and in vitro.

## Materials and methods

### Cell lines

The human epithelial ovarian cancer cell lines SKOV3, A2780, OVCAR3, OVCAR4, IGROV1, ES2 and COC1 were obtained from the Biological Cell Institute of Chinese Peking Union Medical College (Chinese Academy of Medical Science) and cultured in HG-DMEM (Gibco Invitrogen, Carlsbad, CA) supplemented with 10% FBS (HyClone, South Logan, UT) at 37 °C and 5% CO_2_.

### SP cell sorting

When ovarian cancer cells had reached a logarithmic growth phase, they were analysed by Moflo (Beckman Coulter, Fullerton, CA). Cells were digested with 0.25% trypsin (Sigma–Aldrich), washed twice and resuspended in HG-DMEM with 2% FBS at a concentration of 1 × 10^6^ cells/mL. Hoechst 33342 was added at a final concentration of 5 μg/mL, incubated for 90 min in the dark, and then washed twice. The cells were kept at 4 °C, and 1 μg/mL propidium iodide (Sigma–Aldrich) was added before sorting. Verapamil (50 μg/mL, a calcium ion tunnel antagonist) was added for 30 min at 37 °C before adding Hoechst 33342 to control cells, which blocked the fluorescent efflux of SP cells in ovarian cancer cells. SP cells were sorted and cultured in vitro. When SP cells reached a logarithmic growth phase, second SP and non-SP (NSP) sorting was performed to improve the ratio of SP cells.

### Stable isotopic labelling by amino acids in cell culture (SILAC)

The SILAC membrane protein identification and quantitation kit from Invitrogen (Carlsbad, CA) was used for cell labelling and fractionation according to the manufacturer’s instructions. Briefly, SKOV3 and A2780 human epithelial ovarian cancer cells were seeded and cultured in modified DMEM supplemented with 10% dialyzed FBS and either 0.1 mg/ml light L-lysine or heavy [U-13C6] L-lysine. After six doubling times, SP and non-SP cells were harvested and sorted on a Moflo (Beckman Coulter, Fullerton, CA). A total of 106 SP cells labelled with [U-13C6] L-lysine and non-SP cells labelled with light L-lysine were mixed at a 1:1 ratio and lysed in 1.6 ml membrane protein lysis buffer with 0.08% benzonase nuclease on ice for 30 min. The lysate was mixed with 0.4 ml of 1.25 M sucrose solution and centrifuged at 500 g for 10 minutes at 4 °C to remove the nuclear fraction. The supernatant was centrifuged at 100,000 g for 1 hour at 4 °C to obtain the membrane pellet, which was resuspended in 20 μl Nu AGE LDS sample buffer and resolved by SDS–PAGE (dodecyl sulfate, sodium salt-polyacrylamide gel electrophoresis). The entire gel lane was cut into 45 fractions and subjected to in-gel trypsin digestion as described in previous work [15]. Trypsinized peptides labelled with light or heavy L-lysine were analysed using nanoelectrospray LC–MS/MS (liquid chromatograph-mass spectrometer/mass spectrometer) with a Q-TOF API-US mass spectrometer (Waters Corporation, Milford, MA). After candidate peptides were determined with 95% confidence, corresponding proteins were identified using Mascot software with minimum confidence of 80% (Matrix Science, Boston, MA) and validated using immunoblotting with specific antibodies.

### Sphere forming and re-plating assay

Single suspension cells were cultured in ultralow-attachment 6-well plates (Corning, Corning, NY, USA) at 500 ~ 2000 cells/ml sphere culture medium for 2–3 weeks. Once spheres reached approximately 150 μm in diameter, they were digested with Accutase (Invitrogen, Carlsbad, CA, USA) into single cells for replating culture, flow cytometric analysis or sorting. The sphere culture medium was composed of DMEM/F12 (Hank’s medium) (Invitrogen, Carlsbad, CA, USA) 0.4% bovine serum albumin (Sigma, St. Louis, MO, USA), 4 mM L-glutamine, 1 mM sodium pyruvate, 0.1 mM MEM nonessential amino acids, 20 ng/ml recombinant human epidermal growth factor (Invitrogen Carlsbad, CA, USA), 20 ng/ml basic fibroblast growth factor (Invitrogen, Carlsbad, CA, USA), 5 μg/ml insulin, and and 10 ng/ml leukaemia inhibitory factor (LIF, Peprotech, Rocky Hill, NJ).

### Immunofluorescence analysis

To perform immunofluorescence analysis, cells were cultured onto glass slides. After fixation with ice-cold 4% paraformaldehyde for 10 minutes and permeabilization with Triton-X-100 for 30 minutes, cells on the slides were blocked with 3% BSA (bovine serum albumin) for 1 hour and incubated with antibody at 4 °C overnight. Following 5 minutes of washing with PBS 3 times, the slides were incubated with a fluorescein-conjugated IgG antibody (Santa Cruz Biotechnology, CA) in the dark at room temperature for 1 hour. They were also counterstained with propidium iodide for 10 minutes. Confocal images were acquired on a Radiance 2100 confocal laser-scanning microscope (Bio–Rad, Hercules, CA, USA).

### Immunoblotting

Immunoblotting was performed as previously described [16]. The CDC50A antibody used in the study was from Santa Cruz Biotechnology (Santa Cruz, CA, USA). All blots were cut prior to hybridisation with antibodies during blotting.

### Reverse transcription-polymerase chain reaction (RT–PCR)

Total RNA was extracted from cells using the RNA Mini Kit (Qiagen), reverse-transcribed into cDNA, and amplified for 30 cycles in 25 μl reactions with 10 pmol primers The PCR products were electrophoresed in 1% agarose gels. Amplification of β-action was used as a control. Primers for stem cell-associated genes are listed in Supplementary Table 1.

### Quantitative real-time polymerase chain reaction (qRT-PCR)

The extraction of total RNA from cells and synthesis of cDNA have been described on RT-PCR. Specific quantitative real**–**time PCR experiments were performed using PowerUp™ SYBR™ Green Master Mix (Applied Biosystems™).

### Fluorescence-activated cell sorting (FACS) and analysis

Single cells were counted and diluted in HBSS^+^ buffer (1 × HBSS, 2% foetal bovine serum, 10 mM HEPES, pH 7.2, 1% penicillin-streptomycin, Invitrogen, Carlsbad, CA, USA) to obtain up to 10^7^ cells per mL. Each sample was stained at 4 °C with antibody. Primary cells isolated from clinical tumours were also stained with lineage (Lin) markers, including CD235a, CD45, CD31 and CD140a. After washing, the cells were resuspended and sorted on a Moflo Beckman Coulter (Fullerton, CA, USA) or analysed on an LSRII Fortessa (BD Biosciences). Isotype-matched primary and secondary antibodies were used as controls.

### shRNA construction and lentivirus package

Four shRNAs targeting CDC50A were designed and synthesized by GenePharma (Shanghai, China) and cloned into pGPU6/GFP/Neo vectors. After the four shRNA plasmids plus negative control shRNA plasmids were transiently transfected into 293FT cells for 48 hours, the expression of CDC50A was evaluated using Western blotting. Compared with the control shRNA-expressing construct, shCDC50A-974 was the most effective in reducing CDC50A expression (Supplementary Fig. 1). The target sequence of the negative control shRNA was 5′ TTCTCCGAACGTGTCACGT 3′. To generate cells stably expressing the shRNA, shRNA TMEM30A-homo-974 was cloned into the pLKO.1-GFP lentivirus vector, which was then cotransfected with the packaging plasmids pCMV-dR8.91 and pCMV-VSV-G into 293FT cells with PEI transfection reagent. Viruses were harvested 48 hours post transfection and filtered with 0.45 μm syringe filters (Millipore, Milford, MA, USA).

#### Construction of CDC50A expression vector

Primers for CDC50A transcriptional variant 1 (GENE ID: 55754, HUGO Gene Nomenclature Committee) were designed and synthetised (Qiagen, up 5′ -GCGGAATTCGCCACCATGGCGATGAACTATAAC – 3′; down 5′ -GCCGCGGCCGCTTACTTATCGTCGTCATCCTTGTAATCTCCTCCTCCAATGGTAATGTCAGCTG - 3′, 1086 bp). CDC50A gene was amplified (35 cycles, 25 μl reactions with 10 pmol primers) and verified by sequencing. To generate cells stably up-regulated expressing CDC50A, the PCR amplified product was cloned into the pLVX-IRES-GFP virus vector, which was then cotransfected with the packaging plasmids pCMV-dR8.91 and pCMV-VSV-G into HEK-293 T cells. Viruses were harvested 48 hours post transfection and filtered with 0.45 μm syringe filters (Millipore, Milford, MA, USA).

### Mice

All of the procedures involving animals in this study were approved by the Animal Ethics Committee of PUMCH in accordance with institutional and Chinese government guidelines for animal experiments. Nod;Scid (NOD. CB17-Prkdcscid) mice were purchased from the Institution of Laboratory Animal Sciences, Chinese Academy of Medical Sciences, Beijing, China. NSG (NOD. Cg-PrkdCscidIl2rgtm1Wjl/SzJ) mice were obtained from Jackson Laboratory, Bar Harbor, ME, USA. Mice were housed under specific pathogen-free conditions with sterile acidified water and irradiated food. Female mice of 4 to 6 weeks old were used in the study. At least 3 mice were used in each group. Mice were killed by cervical dislocation.

### Tumour xenograft

Cells were diluted and mixed with Matrigel (BD Biosciences, San Jose, CA) at a 1:1 ratio. The mixtures were implanted subcutaneously into the scapular region of mice [17]. Tumour development was monitored by palpation and visual inspection twice a week. Mice were killed and xenograft tumours were harvested for subsequent sorting when tumours were confirmed by palpation or visual. If there were no tumours after 5 months of cell implantation, mice were killed and further determine whether the tumours were formed by anatomy. Tumour tissue was confirmed by immunohistochemistry.

### Tumour tissue

Fresh tumour tissues or ascites were collected from 23 patients with epithelial ovarian cancer from June 2014 to May 2015. Among them, 16 patients were diagnosed with ovarian high-grade serous carcinoma during primary debulking surgery, and their tumour recurrence outcome and PFI were followed. This work conformed to the guidelines explained in the Declaration of Helsinki and was approved (Approval No. S-072) by the Ethics Committee of Peking Union Medical College Hospital (PUMCH, Beijing, China). Informed consent was obtained from all patients and/or their legal guardians. The pathologic diagnosis for all patients was performed by two experienced gynaecologic pathologists. Tumour specimens were analysed with FACS, and CDC50A^+^Lin^−^ cells were counted. PFI was defined as the time interval between the dates of completion of standard platinum-based chemotherapy and that of the first confirmed sign of disease recurrence or the last follow-up.

### Statistical analysis

Data analyses were performed with SPSS 19.0 software. T tests and one-way analysis of variance (ANOVA) were used to compare differences between two study groups. Survival curve was drawn with univariate Cox regression analysis. Hazard ratio (HR) was calculated after adjusting for the optimal debulking surgery. All the tests were two sided, and *p* values less than 0.05 were considered statistically significant.

## Results

### The screening of differentially expressed cellular membrane proteins between SP and non-SP cell through quantitative proteomics

**Fig. 1:**
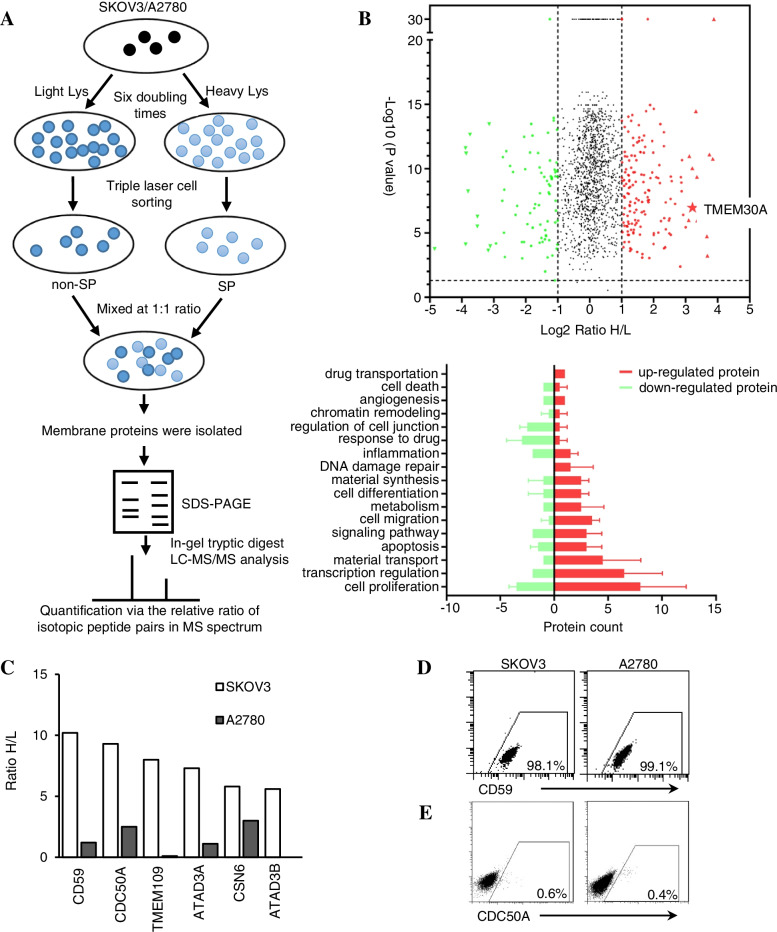
Screening of differentially expressed cellular membrane proteins between SP and non-SP cells through SILAC. **A** Strategy for SILAC is shown. Heavy Lys was used to label proteins in SP cells, and light Lys was used to label proteins in non-SP cells. **B** Differentially expressed proteins in SKOV3 SP cells are shown. Proteins were considered significantly differentially expressed when the *P* value was less than 0.05 and the fold change (ratio H/L) was more than 1. Proteins were classified through protein function. Most of the upregulated proteins had functions of cell proliferation, transcription regulation and material transport. **C.** Six proteins, CD59, CDC50A, TMEM109, ATAD3A, CSN6, and ATAD3B, were found to be differentially expressed in SP cells of SKOV3 through SILAC, and five of them exhibited the same expression difference in SP cells of A2780. CD59 and CDC50A were the two proteins expressed on the cellular surface and were increased in both cell lines. **D, E** Expression of CD59 and CDC50A in SKOV3 and A2780 cells assessed by FACS analysis. CD59 was expressed on nearly all SKOV3 and A2780 cells, whereas CDC50A was expressed on only 0.4–0.6% of them. Based on the reasonable ratio, CDC50A was chosen as the candidate differentially expressed protein in SP cells

Stable isotope labelling with amino acids in cell culture (SILAC) is a robust proteomics technology. In this work, SILAC and LC–MS/MS were used to compare membrane proteins expressed in the SP cells of SKOV3 and A2780 with those in their non-SP cells. The strategy for SILAC is shown in Fig. 1A. Proteins were considered significantly differentially expressed when the *P* value was less than 0.05 and the fold change (ratio H/L) was more than 1. The proteomic data of in this work have been deposited in the OMIX, China National Center for Bioinformation/Beijing Institute of Genomics, Chinese Academy of Sciences (http://ngdc.cncb.ac.cn/omix: accession no. OMIX001138) [18, 19]. In total, 1561 differentially expressed proteins in SKOV3 SP cells were identified, and 77 of the 1561 proteins analysed had an H/L value over 3 (Fig. 1B). In A2780 SP cells, 1989 differentially expressed proteins were detected. All significantly differentially expressed proteins (H/L value > 5) in SKOV3 cells were selected and then referred to the results of A2780 SP cells. Six candidate proteins, TMEM109, ATAD3A, ATAD3B, CSN6, CD59 and CDC50A, increased 5.6–10.2-fold in SP cells (Fig. 1C). Among them, only CD59 and CDC50A were expressed in the cellular surface membrane, while others were expressed in the nuclear membrane or mitochondrial membrane. Notably, CDC50A, not CD59, was selected as a potential surface biomarker for epithelial ovarian cancer-initiating cells because of the more reasonable proportion (nearly 1%, Fig. 1D, E) of positive cells.

### Validation of CDC50A^+^ cell expression in SP ovarian cancer cells

**Fig. 2:**
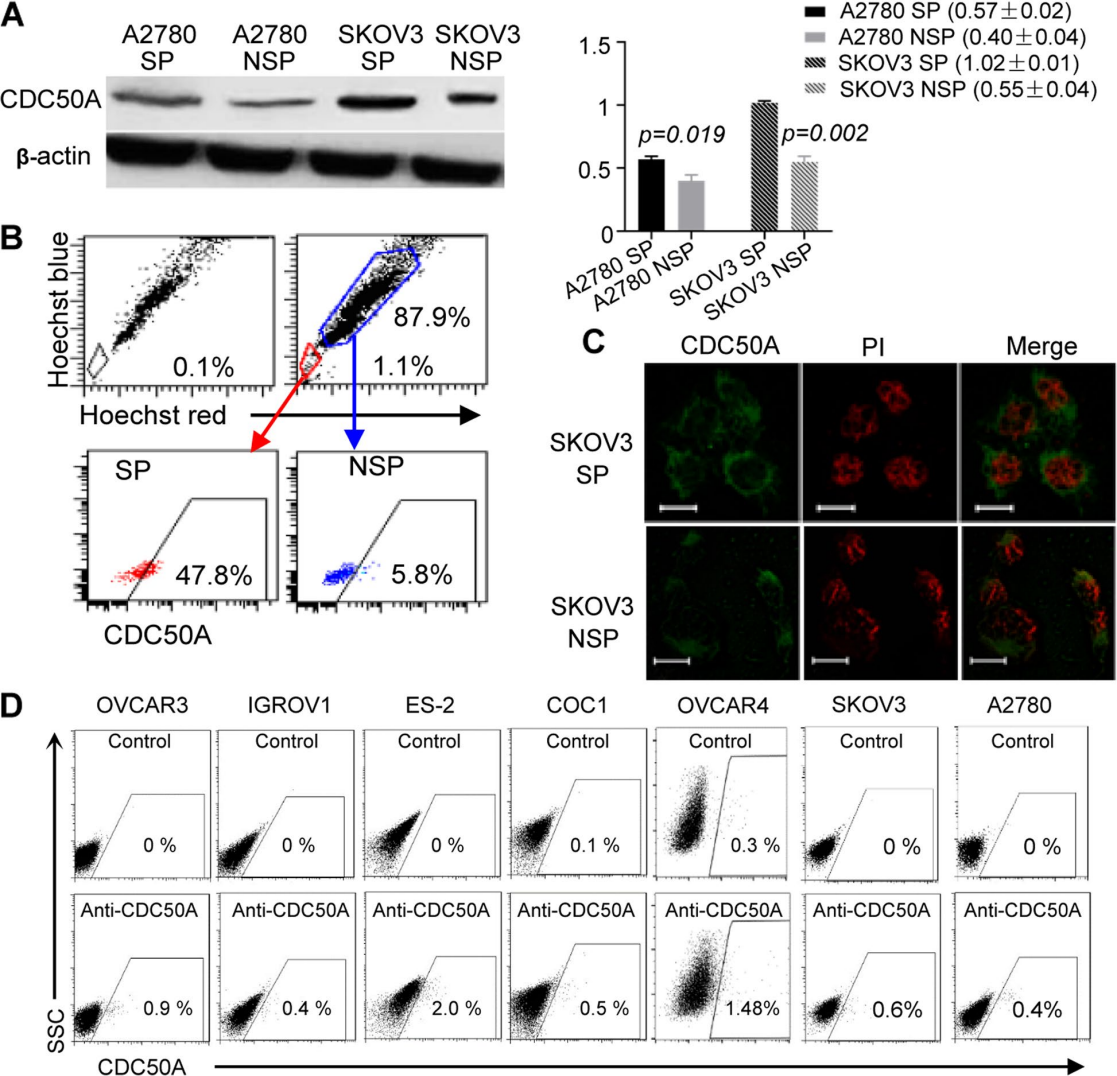
Validation of the differentially expressed cellular surface protein CDC50A in SP cells that might contain cancer initiating cells. **A, B** As detected by western blotting, the expression of CDC50A in SP cells of SKOV3 and A2780 cells increased significantly (*p* = 0.002 and 0.019, respectively). Gray values of bands were measured with ImageJ software (National Institute of Health). Most SP cells were CDC50A-positive cells, as determined by flow cytometry. The grouping of blots cropped from different gels. The blots were cut prior to hybridisation with antibodies. The raw data with detail description was shown in Supplementary Fig. 6. **C** The membrane distribution of CDC50A was validated by immunofluorescence in SP and non-SP SKOV3 cells. Nuclei were stained with PI. Scale bar, 50 μm. **D** The ratios of CDC50A-positive cells in five other ovarian cancer cell lines, in addition to SKOV3 and A2780 cells, were evaluated by flow cytometry. CDC50A-positive cells (0.4% ~ 1.5%) were observed in ovarian cancer cells, which was similar to other common biomarkers of cancer stem cells

First, as evaluated by western blotting (Gray value of bands were measured using ImageJ software), the expression of CDC50A in SKOV3 and A2780 SP cells increased significantly (*p* = 0.002 and 0.019 respectively, Fig. 2A). Compared with non-SP cells, the ratio of CDC50A-positive cells increased significantly in SP cells, as shown by flow cytometry (Fig. 2B). In addition, immunofluorescence analysis revealed that CDC50A was located in the cellular surface membrane. In addition, there was a higher level of CDC50A expression in SP cells than in non-SP cells (Fig. 2C). All of the above results were in accordance with the results of quantitative proteomics.

Furthermore, the ratios of CDC50A-positive cells in five other ovarian cancer cell lines, in addition to SKOV3 and A2780 cells, were evaluated by flow cytometry. CDC50A-positive cells (0.4% ~ 2.0%) were shown in ovarian cancer cells, which was similar to other common biomarkers of cancer stem cells (Fig. 2D). Because of the more reasonable proportion of positive cells in 5 EOC cell lines than in other common CSC biomarkers, such as CD44, CD117 and CD133 (Supplementary Table 2), CDC50A might be a candidate biomarker of epithelial ovarian cancer-initiating cells. Next, the biological characteristics of CDC50A-positive cells were analysed.

### Validation of the biological characteristics of CDC50A-positive EOC cells as cancer-initiating cells: proliferation, self-renewal, differentiation, and tumorigenicity

**Fig. 3:**
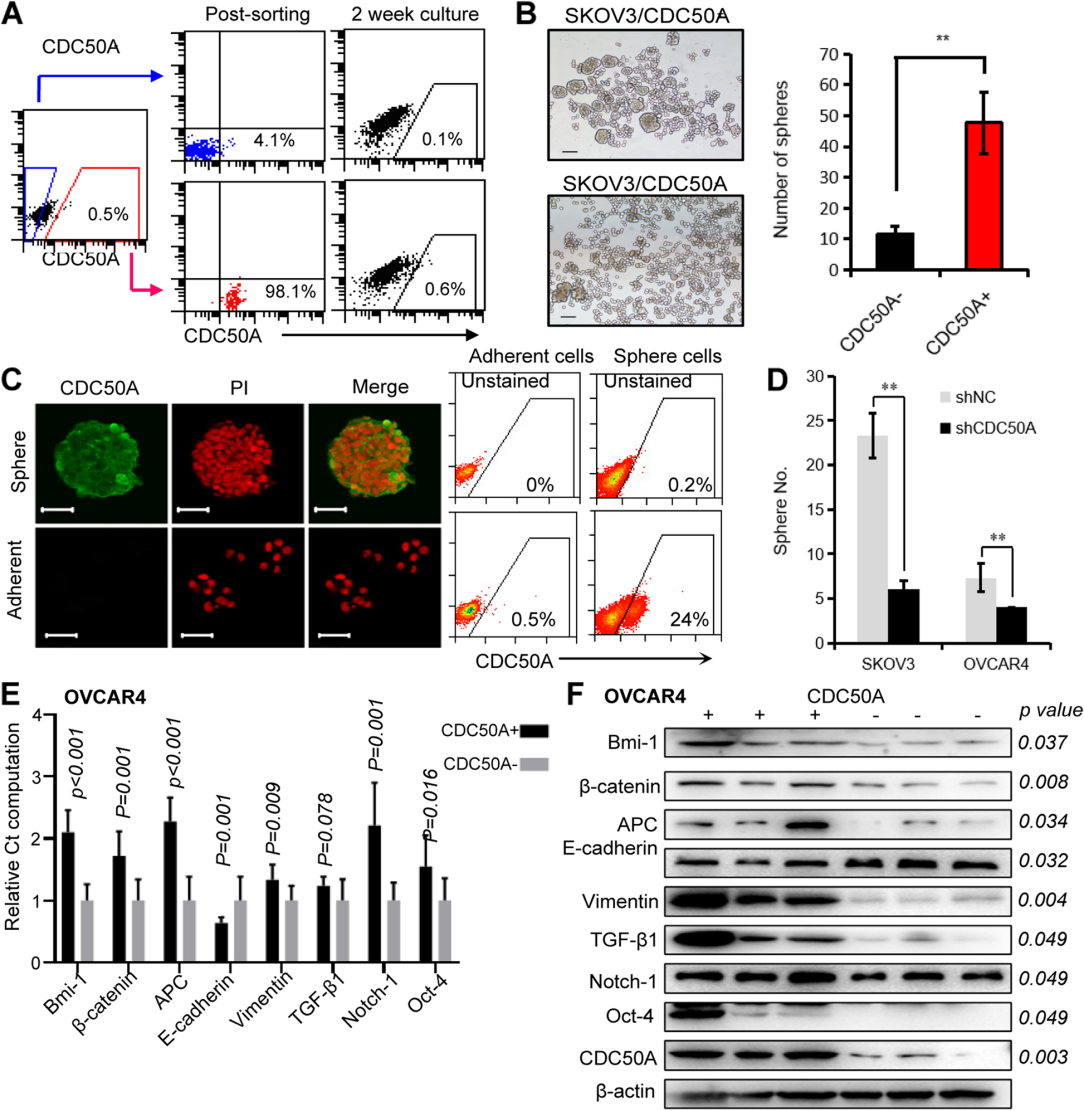
As SP cells, CDC50A-positive SKOV3 cells show similar characteristics to EOC initial cells. **A** CDC50A^+^ SKOV3 cells were sorted and cultured in vitro, and most of them differentiated into CDC50A^−^ cells after 2 weeks, while CDC50A^−^ SKOV3 cells could not generate CDC50A^+^ cells. This demonstrated the ability of CDC50A + SKOV3 cells to differentiate. **B** 1000 cells were cultured for sphere forming. After 7 days, isolated CDC50A^+^ SKOV3 cells generated 2–3 times more spheres than CDC50A^−^ cells (**p < 0.001). CDC50A^−^SKOV3 cells exhibited cell adhesion with each other but not spheres. **C** A higher ratio of CDC50A^+^ SKOV3 cells was detected in spheres than in adherent cultured cells through both immunofluorescence and FACS. Scale bar was 50 μm. **D** The numbers of spheres decreased significantly after the expression of CDC50A was downregulated through shRNA (**p < 0.001). **E** Through qRT-PCR, the mRNA expressions of some stem cell markers, such as Bmi-1, β-catenin, APC, E-cadherin, Vimentin, Notch-1 and Oct-4, increased significantly in CDC50A^+^ OVCAR4 (all *p* < 0.05). **F** The expressions of above stem cell markers were detected using Western-blot, and all of them increased significantly, including TGF-β1 of which the mRNA level was the same between CDC50A^+^ and CDC50A^−^ OVCAR4. Gray value of bands was measured using ImageJ software (National Institutes of Health). The grouping of blots cropped from different gels. The blots were cut prior to hybridisation with antibodies. The images were generated by chemiluminescence image analysis system (Tanon 520, Shanghai, China). The raw data with detail description was shown in Supplementary Figs. 7 and 8

To detect the differentiation ability of CDC50A^+^ cells, after culturing as adherent cells for 2 weeks, the CDC50A^+^ cells (> 97% pure) gave rise to both CDC50A^+^ and CDC50A^−^ cells and further re-established a hierarchy in which the CDC50A^+^ cells reached a minimum proportion of 0.6% of the population. In contrast, few CDC50A^+^ cells appeared in the culture of CDC50A^−^ cells, demonstrating that only CDC50A^+^ cells are capable of differentiating into CDC50A^−^ cells in vitro (Fig. 3A).

A sphere-forming and sphere-replating assay was utilized to assess the self-renewal ability of CDC50A^+^ cells. In this system, isolated CDC50A^+^ SKOV3 cells generated 2–3 times more spheres than CDC50A^−^ cells, and they possessed robust replating activity (Fig. 3B). In addition, cells in spheroids of SKOV3 were largely CDC50A^+^, the proportion of which increased from ~ 0.5% in regular cultures to 24% of the cells in the sphere formation cultures (Fig. 3C). When CDC50A expression in SKOV3 was up-regulated, sphere forming capability was significantly improved (Supplementary Fig. 2).

The tumorigenicity of CDC50A^+^ SKOV3 cells was examined through utilization of the serial xenograft assay in immunocompromised NOD/SCID mice. Inoculation of as few as 10^2^ CDC50A^+^ cells resulted in the formation of tumours in 50% of the mice, whereas no tumours were found after 10^3^ CDC50A^−^ cells were administered (Table 1, Supplementary Figs. 3 and 4).

**Table 1:** Incidence of tumours from SKOV3 cells and primary ovarian cancer cells serially transplanted in NOD/SCID or NSG mice (Tumorigenic and self-renewal activity of CDC50A^+^Lin^−^ and CDC50A^−^Lin^−^ cells sorted from human EOC tumours of 11 patients in NSG mice (11 independent experiments))

		1 × 10^2^ cells		1 × 10^3^ cells		1 × 10^4^ cells		1 × 10^5^ cells	
		1st passage	2nd passage	1st passage	2nd passage	1st passage	2nd passage	1st passage	2nd passage
SKOV3 (NOD/SCID)	CDC50A^+^	2/4	ND	4/4	2/3	4/4	ND	ND	ND
	CDC50A^−^	ND	ND	0/3	0/3	2/6	ND	ND	ND
Primary (NSG)	CDC50A^+^/Lin^−^	ND	ND	1/4	ND	3/8	3/5	4/5	ND
	CDC50A^−^/Lin^−^	ND	ND	0/4	ND	0/8	0/5	1/4	ND

*ND* not detected

Furthermore, when the level of CDC50A expression in CDC50A-positive SKOV3 and OVCAR4 cells was downregulated by shRNA, the number of spheres decreased significantly (*p* < 0.001, Fig. 3D).

In addition, some stem cell-associated genes in CDC50A positive OVCAR4 cells were detected through qRT-PCR and immunoblotting, such as Bmi-1 [20], β-catenin [21], APC [22], E-cadherin [23, 24], vimentin [24, 25], TGF-β1 [26, 27], Notch-1 [28] and Oct-4 [29]. Except TGF-β1, both mRNA and protein level of other 7 genes in CDC50A positive cells increased significantly (all *p* < 0.05, Fig. 3E, F). But the protein expression of TGF-β1 in CDC50A positive OVCAR4 cells was higher than CDC50A negative cells (Fig. 3F).

### CDC50A-positive cells from primary ovarian cancers met the criteria of cancer-initiating cells

**Fig. 4:**
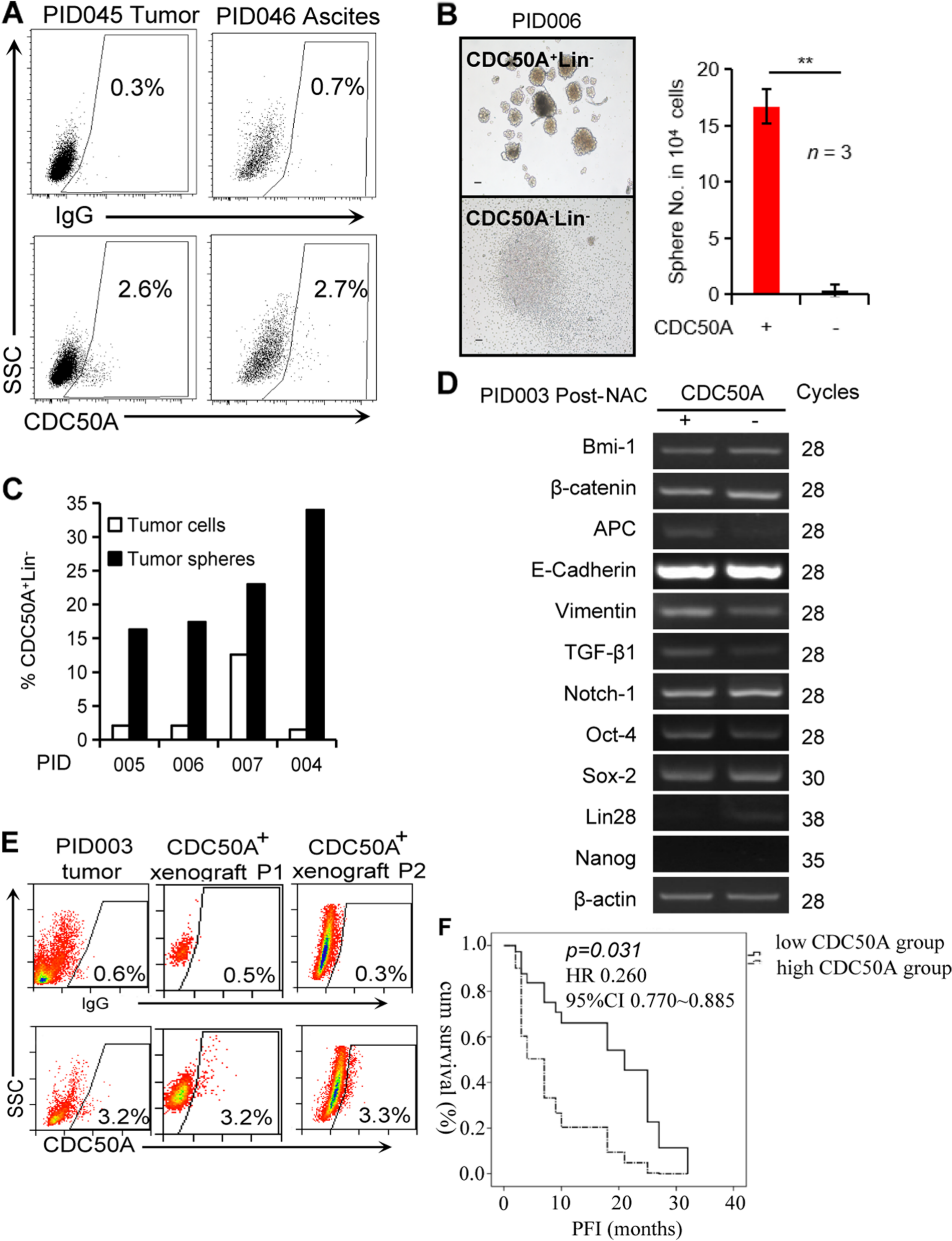
Further validation of CDC50A as a biomarker of cancer-initiating cells in primary epithelial ovarian cancer tissues. **A** CDC50A^+^ cells could be detected through FACS in human ovarian tumours (PID045) and ascites (PID046). **B** shows the much higher sphere-forming activity of CDC50A^+^Lin^−^ cells than CDC50A^−^Lin^−^ cells sorted from individual epithelial ovarian cancer tumours (PID003, PID006 and PID008, input: 10^4^ cells/well). Scale bar, 50 μm. *N* = 3, *P* < 0.001. **C** Higher fractions of CDC50A^+^Lin^−^ cells in sphere culture with cells isolated from epithelial ovarian cancer tumours (4.6% vs. 22.7% on average, *n* = 4). **D** A representative result of semiquantitative PCR analysis of markers for stem cells and epithelial-mesenchymal transition in CDC50A^+^ cells from a primary ovarian cancer (PID003). The grouping of gel cropped from different gels. The raw data was shown in Supplementary Fig. 9. **E** A representative FACS analysis of primary and secondary xenografts in NSG mice generated with CDC50A^+^ cells sorted from human epithelial ovarian cancer (PID003). The percentage of CDC50 A^+^ cells was similar to that of the original cancer. **F.** When the percentage of CDC50A^+^ cells in EOC tissue was over 4.145%, these patients were separated into a high CDC50A group, and the rest were sorted into a low CDC50A group. Between the two groups, after adjusting for the optimal debulking surgery, the number of CDC50A-positive cells was correlated with PFI (*p* = 0.031, HR 0.260, 95% CI 0.770 ~ 0.885)

Twenty-three primary cancer tissues were collected from ovarian cancer patients, and CDC50A^+^Lin^−^ cells were sorted by FACS. Among them, 16 patients received primary debulking surgery followed by platinum-based chemotherapy. All of them had high-grade ovarian serous carcinoma, and the prognoses of the 16 patients were followed. The 6 of remaining 7 patients were treated with neoadjuvant chemotherapy followed by interval debulking surgery. One was recurrent patient and diagnosed with malignant Mullerian epithelial cancer. Three of 7 had carcinoma or clear cell cancer. As shown in Supplementary Table 3 and Fig. 4A, 0.6% ~ 49.5% of CDC50A^+^ cells were found in the Lin^−^ tumour cell population. The ratios of CDC50A^+^ Lin^−^ in the 22 primary ovarian cancer tumours before neoadjuvant chemotherapy ranged from 0.6 to 12.8% (average 9.62%). Ascites from 3 patients were collected, and average 4.97% of CDC50A^+^Lin^−^ cells could be detected in ascites. Their frequency varies significantly with tissue origins, histological type, and chemotherapy (Supplementary Fig. 5).

Isolated CDC50A^+^Lin^−^ cells from primary ovarian tumours were cultured in vitro. While 10^4^ CDC50A^−^Lin^−^ cells formed few spheres in the nonadherent culture, the same number of CDC50A^+^Lin^−^ cells were capable of generating more than 10 spheres (Fig. 4B). CDC50A^+^ cells could be enriched from sphere-forming culture in vitro (Fig. 4C). In addition, the levels of stem cell-associated genes, including β-catenin [21], APC [22], Notch-1 [28], vimentin [24, 25] and TGF-β1 [26, 27], were all increased in CDC50A^+^Lin^−^ cells (Fig. 4D).

The tumorigenicity of CDC50A^+^Lin^−^ cells sorted from ovarian tumours was then assessed using Nod;Scid;IL2rγ^−/−^ (NSG) immunocompromised mice. As shown in Table 1, inoculations of 10^3^, 10^4^ or 10^5^ CDC50A^+^Lin^−^ cells were able to develop xenograft tumours in one-fourth, three-eighth or four-fifth of mice, respectively, whereas only administration of 10^5^ CDC50A^−^Lin^−^ cells was able to generate tumours in a quarter of mice. Notably, only small percentages of the xenograft tumour cells were CDC50A^+^ (Fig. 4E), apparently capitulating to the development of original human tumours. Furthermore, these CDC50A^+^Lin^−^ cells from primary ovarian tumours could be sorted and passaged in NSG recipient mice (Table 1, Supplementary Figs. 3 and 4). Taken together, these data demonstrated that CDC50A^+^Lin^−^ cells from primary ovarian tumours have the ability to self-renew and differentiate in vivo.

### High levels of CDC50A in ovarian cancer tumours might be correlated with poor prognosis

The ratios of CDC50A-positive cells in primary high-grade ovarian serous carcinoma tissues from the 16 patients described above were analysed through FACS, and clinical prognosis was assessed. Disease recurrence were the primary terminal. The percentage of CDC50A-positive cells in primary cancer tissues ranged from 0.6 to 7.4%. Among these 16 patients, 5 patients were platinum resistant, and the remaining 11 were platinum sensitive. When the 50th percentile (4.145%) was considered the cut-off value of the high CDC50A group and low CDC50A group, there were different PFIs between the two groups. After adjusting for the optimal debulking surgery, CDC50A-positive cells were significantly correlated with poor prognosis by Cox regression analysis (*p* = 0.031, HR 0.260, 95% CI 0.77 ~ 0.885, Fig. 4F).

### CDC50A^+^Lin^−^ cells isolated from primary ovarian cancers exhibited characteristics of mesenchymal transition (EMT)

**Fig. 5:**
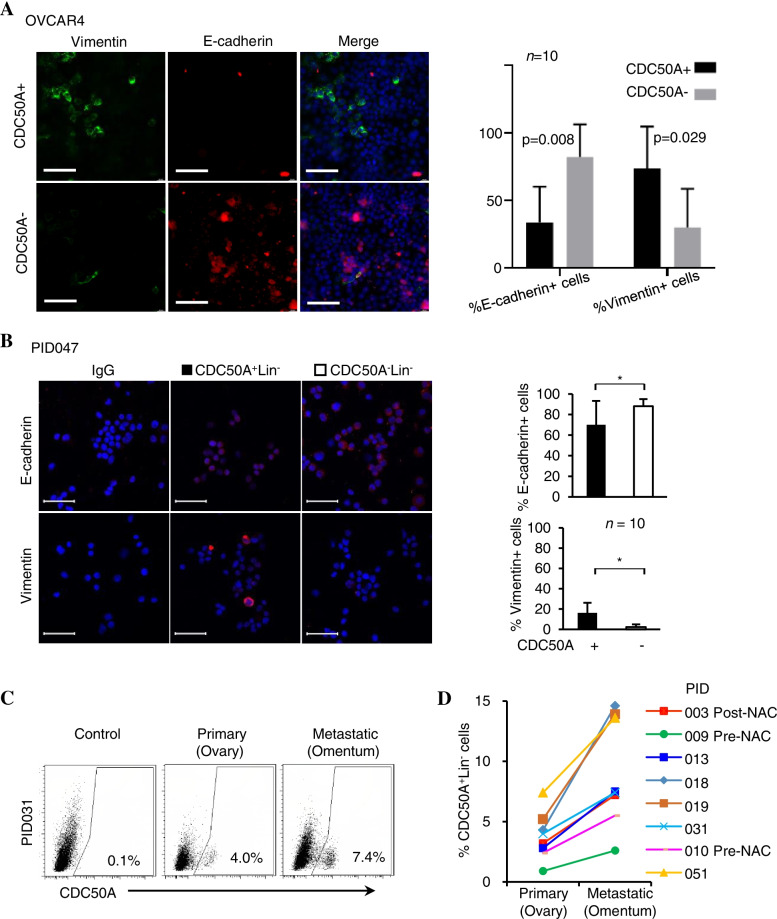
Association of CDC50A+ cells with epithelial-mesenchymal transition (EMT) and metastasis. **A** E-cadherin and vimentin expression in CDC50A^**+**^ OVCAR4 and CDC50A^−^ OVCAR4 cells was detected through immunofluorescence. Compared with CDC50A^−^ OVCAR4 cells, the level of E-cadherin decreased significantly in CDC50A^+^ OVCAR4 cells (33.4% vs. 82.2%, *p* = 0.008), and vimentin increased (73.7% vs. 29.8%, *p* = 0.029). E-cadherin is shown in red (middle), and vimentin (left) is shown in green. Nuclei were stained with DAPI in blue (right). Scale bar, 50 μm. The right panel shows the mean percentages of positive cells quantified from 10 confocal images. **B** The EMT status of sorted CDC50A + Lin- and CDC50A-Lin-cells expressing E-cadherin (upper left panel, red, 70.1% vs. 88.0%) and vimentin (lower left panel, red, 16.2% vs. 2.0%) in an EOC tumour from patient PID047. Nuclei were stained with DAPI in blue. Scale bar, 50 μm. The right panel shows the mean percentages of positive cells quantified from 10 confocal images. **C** Through FACS, CDC50A + Lin-populationin was higher in metastatic (omentum) than in situ ovarian tumours (PID031). **D** shows consistently higher frequencies of CDC50A + Lin- cells in metastatic tumours from the omentum than corresponding ovarian tumours in situ (*n* = 8)

It has been reported that epithelial mesenchymal transition (EMT) plays an important role in tumour metastasis and that tumour cells with EMT have stem cell properties [24]. As shown in Supplementary Table 3, the frequency of CDC50A^+^Lin^−^ cells was associated with the dissemination and metastasis of ovarian tumours. Immunostaining with antibodies against E-cadherin and vimentin (Fig. 5A) revealed that approximately 33.4% of CDC50A positive OVCAR4 were positive for E-cadherin, and 73.7% positive for vimentin. In CDC50A negative OVCAR4, the ratio of E-cadherin positive cells increased significantly (82.2%, *p* = 0.008) and cells positive for vimentin decreased (29.8%, *p* = 0.029). Furthermore, approximately 29.9% of the CDC50A^+^Lin^−^ cells sorted from a relapsed Mullerian tumour (Patient 047) were negative for the epithelial marker E-cadherin, and 16.2% of them were positive for the mesenchymal marker vimentin (Fig. 5B). In contrast, only a few of the CDC50A^−^Lin^−^ cells (2.3%) were vimentin positive, suggesting that CDC50A^+^ cells are more mesenchymal-like and may participate in the dissemination and metastasis of ovarian cancers.

To further explore the relationship between CDC50A^+^Lin^−^ cells and tumour metastasis, both metastasized tumours on the omentum cake and their primary tumours, which originated from the ovarian surface epithelium, were collected from 8 stage III patients. The frequencies of CDC50A^+^Lin^−^ cells were significantly higher in the metastatic tumours than in the corresponding primary ovarian tumours (Fig. 5C, D). Thus, CDC50A^+^Lin^−^ cells are increased both in tumours after neoadjuvant chemotherapy and in metastasized tumours. It is of great interest to further directly determine whether they have an increased ability to disseminate or metastasize.

## Discussion

Over the past decade, the critical role of a small subset of tumour cells, known as CSCs, was established in tumour relapse and propagation. Conventional anticancer therapies, including debulking surgery, chemotherapy and radiotherapy, kill most tumour masses, resulting in tumour shrinkage. However, CSCs differentiate into tumour cells and are responsible for tumour relapse. Targeting the putative CSCs is considered a promising way to improve the outcomes of patients with advanced-stage ovarian cancers. This was the first study to screen and validate a special biomarker of epithelial ovarian cancer-initiating cells. Very few cells were selected and proven to be correlated with tumour development, metastasis and poor prognosis.

The small SP population from ovarian cancer cells has an important role in tumorigenesis and drug resistance [30]. In addition to ABCG2/BCRP1, many embryonic stem cell markers, such as NANOG, OCT4 and STELLAR, were expressed in SP cells of ovarian cancer. Based on a comparison of quantitative proteomics (SILAC) between SP cells and NSP cells of EOC cell lines, CDC50A was screened and validated. In this work, CDC50A was located at the cell membrane of ovarian cancer cells. Higher expression of CDC50A was detected in SP ovarian cancer cells, which were rich in cancer initiation ability. Both in vitro and in vivo, it was confirmed that CDC50A^+^ cells meet the criteria of cancer stem cells, such as proliferation, self-renewal, differentiation, tumorigenicity and tumour metastasis. These biological behaviours of CDC50A^+^ ovarian cancer cells were consistent with those of ovarian cancer cells with positive expression of several classic stem cell surface biomarkers, such as CD44 [31], CD117 [32], CD24 [33] and CD133 [34].

CDC50A belongs to the CDC50 family of membrane proteins, which carry two transmembrane segments with short N- and C-cytoplasmic regions and a large extracellular loop [35] and have been proposed to be a β-subunit of the flippase complex. CDC50A, combined with P4-ATPases, can induce functional lipid flipping, and play important role in maintenance of cell membrane asymmetry by flippase [36]. The function of CDC50A remains largely unexplored. CDC50A might be related with angiogenesis process [37]. It has been reported that CDC50A plays a major role in cell migration. Overexpression of CDC50A induced extensive cell spreading and greatly enhanced cell migration [38]. Interestingly, CDC50A has also been reported to play a role in the uptake of the anticancer drug perifosine in human carcinoma. Overexpression and knockdown of the human beta subunit CDC50A in KB cells enhanced and decreased, respectively, the uptake of both fluorescent aminophospholipid analogues and the anticancer alkylphospholipid perifosine [39]. In addition, overexpression of CDC50A also conferred resistance to oxaliplatin in colorectal cancer patients [40]. CDC50A loss-of-function mutations were associated with favourable outcomes uniquely observed in diffuse large B-cell lymphoma. CDC50A loss-of-function increases the accumulation of chemotherapy drugs and tumour-associated macrophages and the effect of anti-CD47 blockade, limiting tumour growth [41]. It was also reported that CDC50A plays a critical role in the survival of haematopoietic stem cells, as conditional deletion of the molecule resulted in depletion of haematopoietic stem cells and peripheral blood cells [42]. For the first time, it was validated that CDC50A might be correlated with ovarian cancer development.

EMT is a process in which epithelial cells become mesenchymal stem cells. TGF-β1, a potent inducer of EMT, increased in both CDC50A^+^ Lin^−^ cells in primary high-grade serous cancer tissues and OVCAR4. EMT endows cells not only migratory and invasive characteristics but also stem cells properties. Increased EMT markers is closely connected with the emergence of cancer stem cells [43]. Further research is needed to detect the mechanism of TGF-β1 in CDC50A^+^ ovarian cancer cells. Notably, a high ratio of CDC50A-positive cells in primary tumour tissues was correlated with poor prognosis in the clinic. CDC50A could be used as a biomarker of ovarian cancer-initiating cells and might be a novel target to promote prognosis.

However, the whole work was limited to several epithelial ovarian cancer cell lines and few primary tumour tissues, and drug sensitivity has not yet been followed up on. Many questions remain unanswered. First, do primary CDC50A-positive cells show significant drug resistance, and can CDC50A-positive cells escape chemotherapy in real patients? Second, if all is known about the tumour heterogeneity of ovarian cancer, why does the ratio of CDC50A-positive cells vary so significantly among different tissues? Are they pre-existing in the tumours or induced upon repeated chemotherapy? Third, can CDC50A-positive cells be the reason for tumour recurrence? Finally, the mechanism by which CDC50A regulates ovarian cancer cell proliferation and metastasis is still unknown.

## Conclusions

Based on the work reported above, this is the first study to prove that CDC50A-positive epithelial ovarian cancer cells possess properties of ovarian cancer stem cells, including proliferation, self-renewal, differentiation, and metastasis. In addition, CDC50A is also a functional protein that regulates cell proliferation and might be related with oncologic prognosis in clinic. These results indicate that CDC50A could be used to screen ovarian CSCs for further studies on occurrence and development of ovarian cancer and may also serve as a molecular target protein.

## Supplementary Information

**Additional file 1: Supplementary Figure 1.** Screening of shRNAs targeting CDC50A. **Supplementary Figure 2.** Validation of CDC50A expression and location after transfection with pLVX-CDC50A-GFP viruses in SKOV3. Sphere forming test in SKOV3 with up-regulated CDC50A. **Supplementary Figure 3.** All mice and xenografts pictures. **Supplementary Figure 4.** Xenograft tumor tissues (PID003) was confirmed by immunohistochemistry**. Supplementary Figure 5.** The frequency of CDC50A + Lin- cells in clinical patients. **Supplementary Table 1.** List of gene-specific primer sequences. **Supplementary Table 2.** The positive ratio of CDC50A, CD44, CD117 and CD133 in five EOC cell lines. **Supplementary Table 3.** Patient clinical records and frequency of CDC50A^+^Lin^−^ cells. **Supplementary Figure 6.** The raw data of Fig. 2, panel A. The region used in Fig. 2 was marked with red box. Chemiluminescence strip was exposed on Kodak film. Then the image was generated by camera. The blots were cut prior to hybridisation with antibodies. The edge of all images have been exhibited. **Supplementary Figure 7.** The raw data of Fig. 3, panel F. The region used in Fig. 3F was marked with red box. The left three samples in red box were CDC50A+, the right three were CDC50A-. Marker was sampled on both sides of all samples. Gel was cut before protein transfer membrane. Middle lines of the markers were the both sides of gel. The blots were cut prior to hybridisation with antibodies. The images were generated by chemiluminescence image analysis system (Tanon 520, Shanghai, China). The right was the chemiluminescence exposure image. The left was the merge of daylight image with edge of blots and chemiluminescence exposure image. **Supplementary Figure 8.** The raw data of Fig. 3, panel F. The region used in Fig. 3F was marked with red box. The left three samples in red box were CDC50A+, the right three were CDC50A-. Marker was sampled on both sides of all samples. Gel was cut before protein transfer membrane. Middle lines of the markers were the both sides of gel. The blots were cut prior to hybridisation with antibodies. The images were generated by chemiluminescence image analysis system (Tanon 520, Shanghai, China). The right was the chemiluminescence exposure image. The left was the merge of daylight image with edge of blots and chemiluminescence exposure image. **Supplementary Figure 9.** The raw data of Fig. 4, panel D. The region used in Fig. 3F was marked with red box. The left band of Bmi-1, β-catenin, APC, Oct-4, Sox-2, Nanog, E-Cadherin, Vimentin, Notch-1, Lin28 and β-actin were CDC50A+, and the right was CDC50A-. The left band of TGF-β1 was CDC50A- and the right was CDC50A+. **Supplementary Figure 10.** The raw data of Supplementary Fig. 1. The image is generated by the chemiluminescence imaging system (Tanon 520, Shanghai, China). The blots were cut prior to hybridisation with antibodies. The whole raw images have been exhibited. The region used in Supplementary Fig. 1 was marked with red box. The edges of the blots (β-actin) in chemiluminescence exposure image were outlined with solid black lines. **Supplementary Figure 11.** The raw data of Supplementary Fig. 2, panel B. Chemiluminescence strip was exposed on Kodak film. The image was generated by camera. The blots were cut prior to hybridisation with antibodies. The edge of all images have been exhibited. The region used in Supplementary Fig. 2B was marked with red box.

## Data Availability

The proteomic data generated and analysed during the current study are available in the OMIX, China National Center for Bioinformation/Beijing Institute of Genomics, Chinese Academy of Sciences (http://ngdc.cncb.ac.cn/omix: accession no. OMIX001138) [18, 19]. The other data that support the findings of this study are included in this article.

## Abbreviations

EOC: Epithelial ovarian cancer
EMT: Mesenchymal transition
PFI: Platinum-free interval
CSCs: Cancer stem cells
SP: Side population
FACS: Fluorescence activated cell sorting
ABC: ATP-binding cassette
SILAC: Stable isotope labelling with amino acids in cell culture
RT-PCR: Reverse transcription-polymerase chain reaction
qRT-PCR: Quantitative real-time polymerase chain reaction
