# Supplementary material for: CDC50A might be a novel biomarker of epithelial ovarian cancer-initiating cells
Source: BMC Cancer. 2022 Aug 19;22:903. doi: 10.1186/s12885-022-09953-y (PMC9389740; doi:10.1186/s12885-022-09953-y)
Supplement: Supplementary file 1 — Additional file 1: Supplementary Figure 1. Screening of shRNAs targeting CDC50A. Supplementary Figure 2. Validation of CDC50A expression and location after transfection with pLVX-CDC50A-GFP viruses in SKOV3. Sphere forming test in SKOV3 with up-regulated CDC50A. Supplementary Figure 3. All mice and xenografts pictures. Supplementary Figure 4. Xenograft tumor tissues (PID003) was confirmed by immunohistochemistry. Supplementary Figure 5. The frequency of CDC50A + Lin- cells in clinical patients. Supplementary Table 1. List of gene-specific primer sequences. Supplementary Table 2. The positive ratio of CDC50A, CD44, CD117 and CD133 in five EOC cell lines. Supplementary Table 3. Patient clinical records and frequency of CDC50A+Lin− cells. Supplementary Figure 6. The raw data of Fig. 2, panel A. The region used in Fig. 2 was marked with red box. Chemiluminescence strip was exposed on Kodak film. Then the image was generated by camera. The blots were cut prior to hybridisation with antibodies. The edge of all images have been exhibited. Supplementary Figure 7. The raw data of Fig. 3, panel F. The region used in Fig. 3F was marked with red box. The left three samples in red box were CDC50A+, the right three were CDC50A-. Marker was sampled on both sides of all samples. Gel was cut before protein transfer membrane. Middle lines of the markers were the both sides of gel. The blots were cut prior to hybridisation with antibodies. The images were generated by chemiluminescence image analysis system (Tanon 520, Shanghai, China). The right was the chemiluminescence exposure image. The left was the merge of daylight image with edge of blots and chemiluminescence exposure image. Supplementary Figure 8. The raw data of Fig. 3, panel F. The region used in Fig. 3F was marked with red box. The left three samples in red box were CDC50A+, the right three were CDC50A-. Marker was sampled on both sides of all samples. Gel was cut before protein transfer membrane. Middle lines of the mark [file 12885_2022_9953_MOESM1_ESM.pdf]

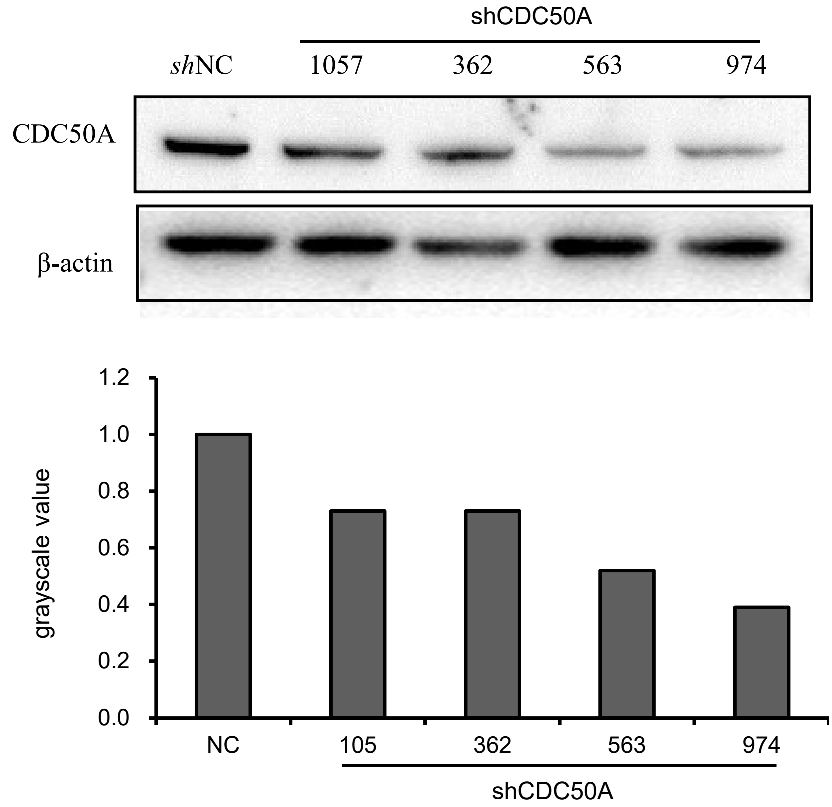

Supplementary Figure 1. Four shRNA targeting CDC50A were designed and synthesized by GenePharma (Shanghai, China) and cloned into pGPU6/GFP/Neo vectors. After the four shRNA plasmids plus negative control shRNA plasmids were transfected into 293FT cells transiently for 48 hours, the expression of CDC50A was examined by using Western blotting. Compared with control shRNA-expressing construct, shCDC50A-974 was the most effective in reducing CDC50A expression. β-actin was used as control. The grouping of blots were cropped from different blots. The blots cut were prior to hybridisation with antibodies. The edge of all images have been exhibited in the raw data (Supplementary Figure 10).

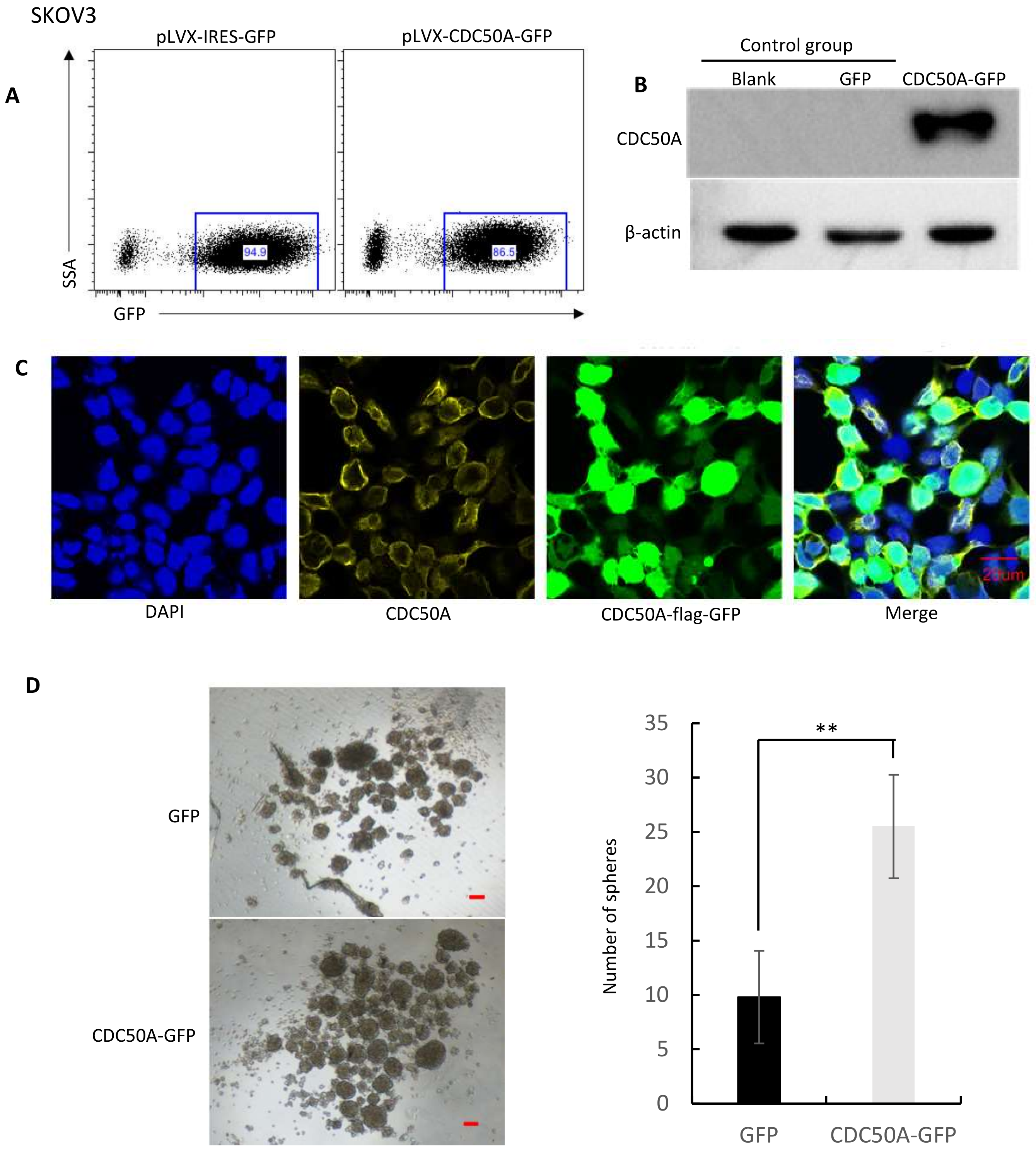

Supplementary Figure 2. A. CDC50A gene was cloned into the pLVX-IRES-GFP virus vector. The pLVX-CDC50A-GFP viruses were packaged and harvested. Viruses were transfected into SKOV3. Through FACS, the transfection efficiencies of pLVX-IRES-GFP and pLVX-CDC50A-GFP were 94.9% and 86.5%. **B.** The expression of CDC50A in SKOV3 could be increased significantly at protein level (Western blot). The grouping of blots were cropped from different blots. The blots cut were prior to hybridisation with antibodies. The edge of all images have been exhibited in the raw data (Supplementary Figure 11). **C.** Immunofluorescence analysis was used to detect the CDC50A location. After transfection with pLVX-CDC50A-GFP in SKOV3, CDC50A could successfully located in the cell surface membrane. **D.** When CDC50A expression in SKOV3 was up-regulated, sphere forming capability was significantly improved. **p<0.001. Bar, 50 µm.

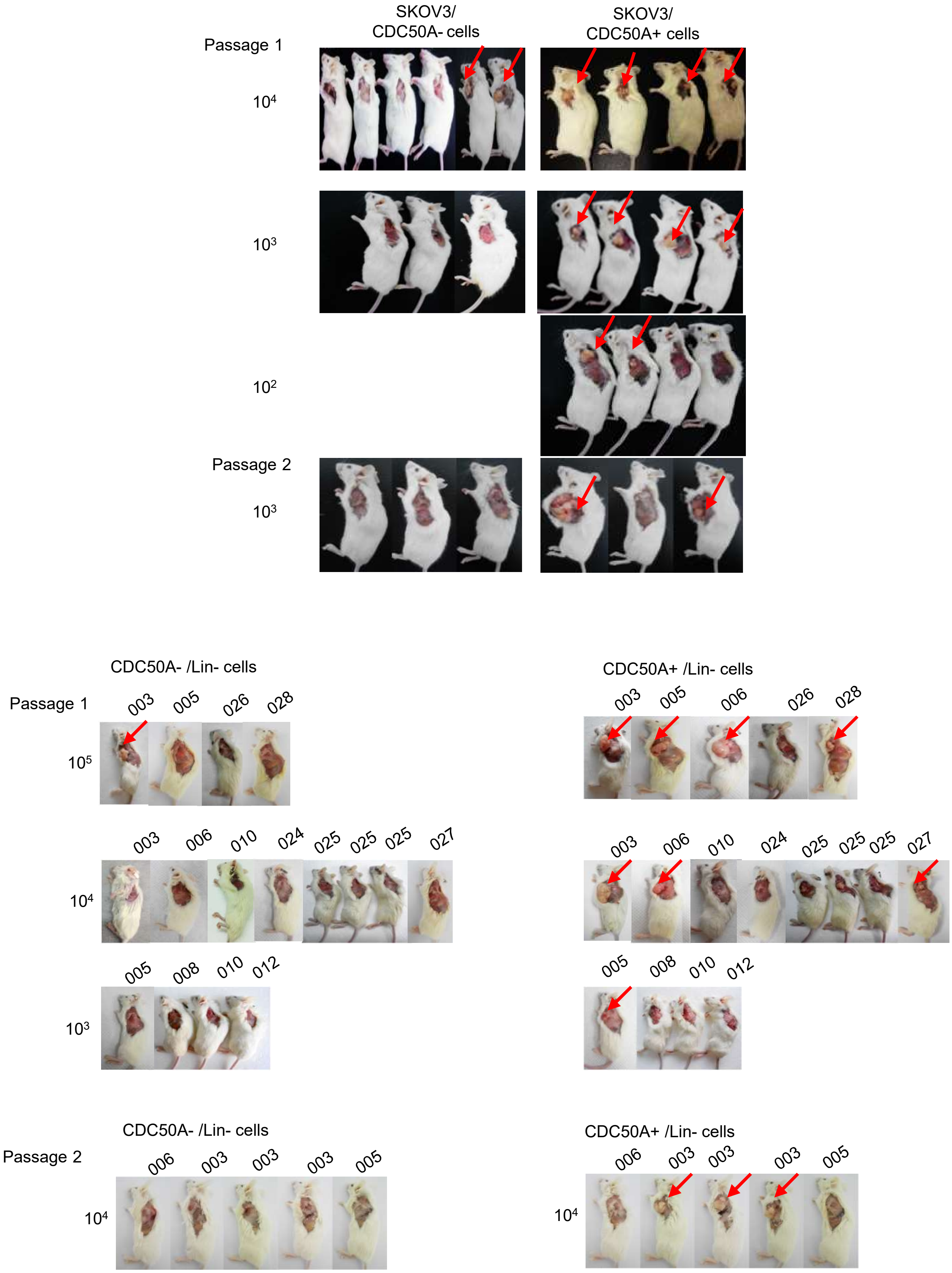

Supplementary Figure 3. The raw data of Table 1. Red arrows marked tumors.

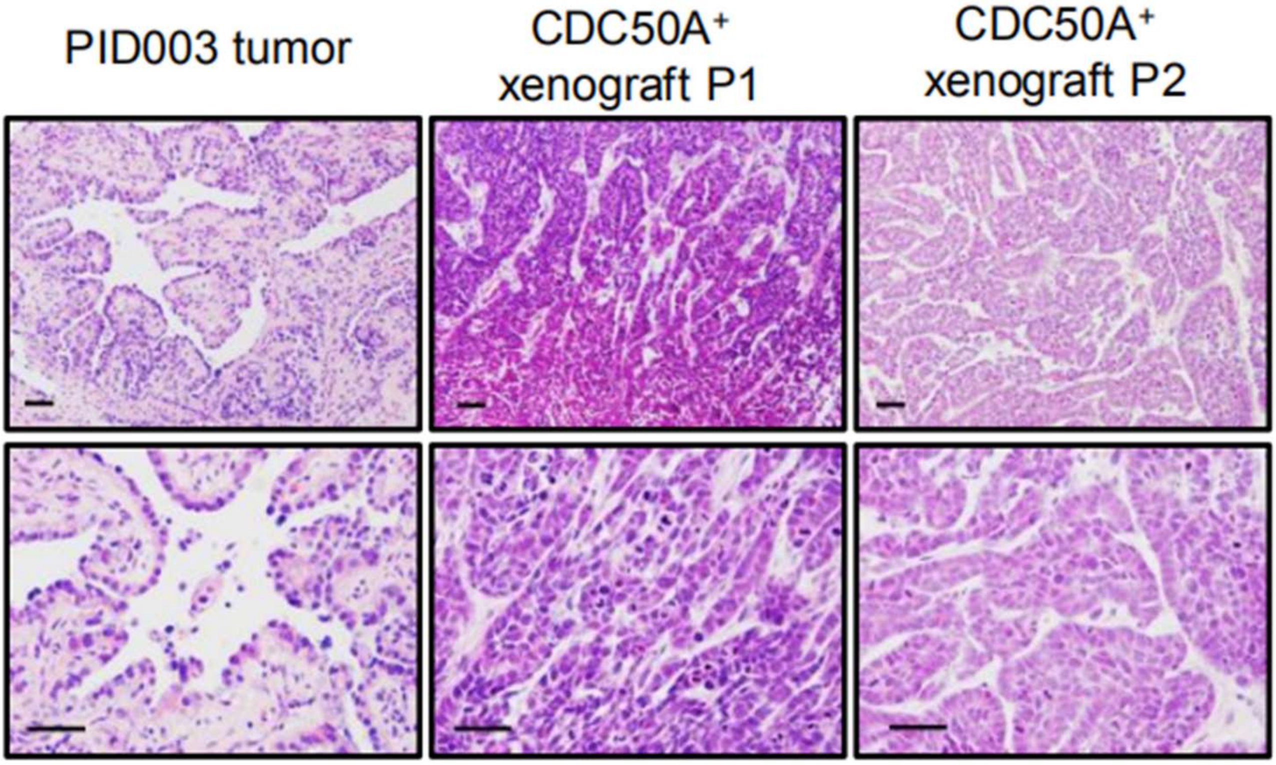

Supplementary Figure 4. Xenograft tumor tissues (PID003) was confirmed by immunohistochemistry**.**

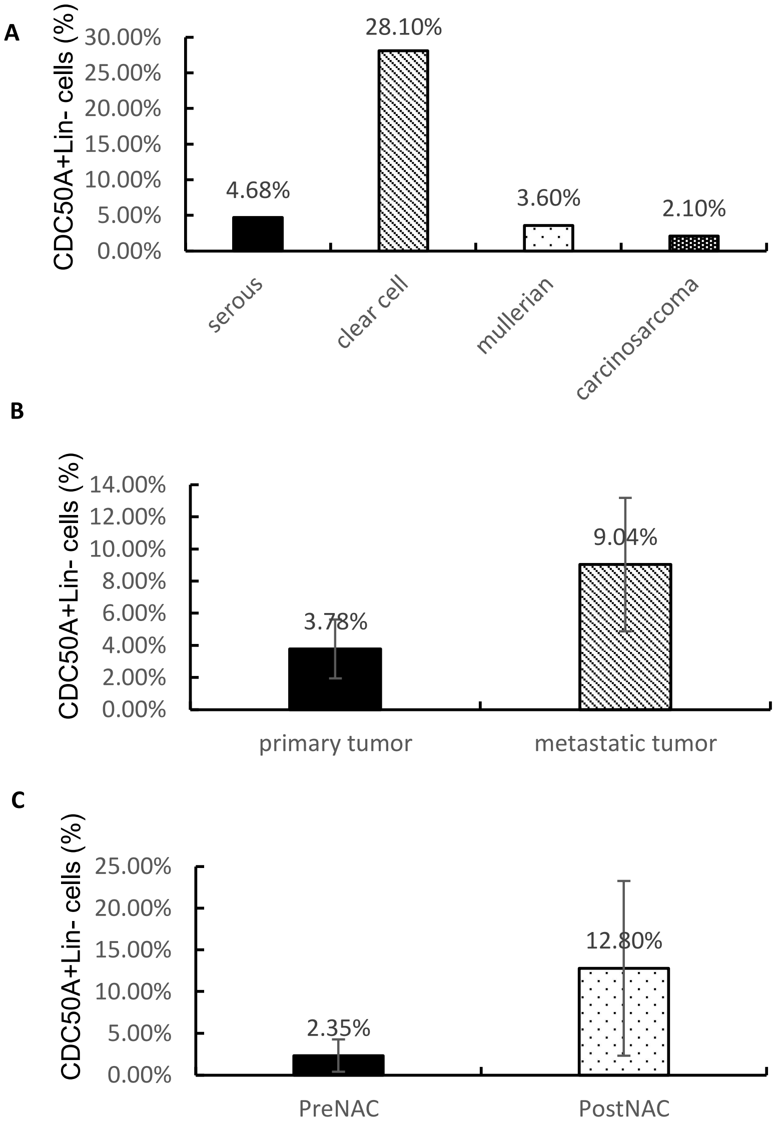

Supplementary Figure 5. The frequency of CDC50A+Lin- cells in clinical patients. **A.** Ratios of CDC50A+Lin- cells in 22 fresh tumor tissues collected during debulking surgery before chemotherapy were shown and varied with histological type. Among them, 18 patients was high-grade serous carcinoma, 2 clear cell carcinoma, 1 Mullerian epithelial carcinoma and 1 carcinosarcoma. **B.** Both primary ovarian cancer tumor and metastatic tumor during debulking surgery before chemotherapy were collected in 8 patients with high-grade serous carcinoma. The average frequencies of CDC50A+Lin- cells in primary tumors and metastatic tumors were 3.76% and 9.04%, respectively. **C.** PreNAC: ovarian cancer tissues were collected from patients before neoadjuvant chemotherapy (NAC); PostNAC: ovarian cancer tissues from patients after NAC. PreNAC and PostNAC of 4 patients with high-grade serous carcinoma were analyzed. The ratios of CDC50A+Lin- cells in PreNAC and PostNAC were 2.35% and 12.80%.

Supplementary table 1. List of gene-specific primer sequences

Gene	Primer	Product	Tm	Cycle
Bmi1	AGATACTTACGATGCCCAGCAG	246bp	60℃	28
	CAGTCCATCTCTCTGGTGACTG			
β-catenin	GAAACGGCTTTCAGTTGAGC	196bp	60℃	28
	TTCCATCATGGGGTCCATAC			
APC	AAATGAGGACCACAGGCAAATC	123bp	60℃	28
	GCTGAGAGATTCCACAAAGTTCC			
E-cadherin	TGGAACAGGGACACTTCTGC	217bp	60℃	28
	TTCTTGGGTTGGGTCGTTGT			
Vimentin	TCTGGATTCACTCCCTCTGG	213bp	61℃	28
	GCAGAAAGGCACTTGAAAGC			
TGFB1	ACATTGACTTCCGCAAGGAC	262bp	60℃	28
	GAGCGCACGATCATGTTG			
Notch1	ATCGATGGCTACGAGTGTGC	240bp	60℃	28
	CTTGTACCCGTTGAGGCTGT			
Lin28	TCGGACTTCTCCGGGGCCAG	107bp	65℃	38
	GCGCAGCCACCTGCAAACTG			
Oct-4	AGGATCACCCTGGGATATACAC	241bp	62℃	28
	TACTGGTTCGCTTTCTCTTTCG			
Sox2	GGGAAATGGGAGGGGTGC	150bp	62℃	30
	TGCGTGAGTGTGGATGGG			
Nanog	ATAGCAATGGTGTGACGCAG	137bp	60℃	35
	CCAGGTTGAATTGTTCCAGGTC			

Supplementary table 2. The positive ratio of CDC50A, CD44, CD117 and CD133 in five EOC cell lines

Cells	CDC50A	CD133	CD44	CD117
SKOV3	1.60±0.04	0.05±0.01	100.00±0.00	0.16±0.01
A2780	1.70±0.04	0.04±0.00	0.29±0.05	0.18±0.06
IGROV1	1.10±0.04	14.00±0.06	84.00±0.35	4.90±0.11
OVCAR3	1.10±0.01	4.10±0.08	91.00±0.20	0.10±0.01
ES2	1.60±0.04	0.08±0.02	100.00±0.00	0.55±0.07

Supplementary table 3. Patient clinical records and frequency of CDC50A+Lin- cells

Patient ID	CDC50A%	Histology	Differentiation	FIGO stage	surgical satisfaction	chemotherapy regimen	platinum sensitivity	PFI (months)
003-PreNAC*	P:0.6% A:8.0%	Serous	Poor	Ⅲ C	suboptimal	TC	sensitive	18
003-PostNAC*	P:3.2%`M:7.2%	Serous	Poor	Ⅲ C				
004	1.5%	Serous	Poor	Ⅲ C				
005	2.1%	Serous	Poor	Ⅲ C				
006	2.1%	Carcinosarcoma	Poor	Ⅳ				
007	12.6%	Serous	Poor	Ⅲ C				
009-PreNAC*	P:0.9% M:2.6%	Serous	Poor	Ⅲ C	suboptimal	TC	sensitive	9
009-PostNAC*	3.5%	Serous	Poor	Ⅲ C				
010-PreNAC	P:2.4% M:5.5%	Serous	Poor	Ⅲ C	suboptimal	TC	sensitive	7
010-PostNAC	15.9%	Serous	Poor	Ⅲ C				
011-PreNAC	P:5.5% A:4.2%	Serous	Poor	Ⅲ C	optimal	TC	sensitive	10
011-PostNAC	28.6%	Serous	Poor	Ⅲ C				
012*	2.8%	Serous	Poor	Ⅲ C	suboptimal	TC	sensitive	25
013*	P:2.8% M:7.5%	Serous	Poor	Ⅲ C	optimal	TC	sensitive	32
014	8.5%	Serous	Poor	Ⅲ C	optimal	TC	resistant	3
016	12.8%	Serous	Poor	Ⅲ C	suboptimal	TC	resistant	4
018*	P:4.3% M:14.6%	Serous	Poor	Ⅲ C	optimal	TC	sensitive	21
019*	P:5.2% M:13.9%	Serous	Poor	Ⅲ C	suboptimal	TC/TP	sensitive	18
029	2.6%	Serous	Poor	Ⅲ B	optimal	TC/TP	sensitive	25
031	P:4.0% M:7.4%	Serous	Poor	Ⅲ C	suboptimal	TC	sensitive	27
038	5.7%	Serous	Poor	Ⅲ C	optimal	TC	resistant	2
039	6.7%	Clear cell	Poor	Ⅲ C				
045	2.6%	Serous	Poor	Ⅲ C	optimal	CC/C	resistant	2
046	A:2.7%	Serous	Poor	Ⅳ	suboptimal	TC	resistant	2
047	R:3.6%	Mullerian	Poor	Ⅱ C				
051	P:7.4% M:13.6%	Serous	Poor	Ⅲ C	optimal	TC	sensitive	7
056	49.5%	Clear cell	Poor	Ⅲ C				

PreNAC: tumors were collected from patients before neoadjuvant chemotherapy (NAC); PostNAC: tumors from patients after NAC. A: ascites; P: tumors collected from the ovary; M: metastatic tumors from the omentum; R: tumors collected from recurrence patients. * indicates: flow cytometry analysis without 7AAD. TC: taxol and carboplatin; TP: taxol and cisplatin; CC: cyclophosphamide and carboplatin; C: carboplatin

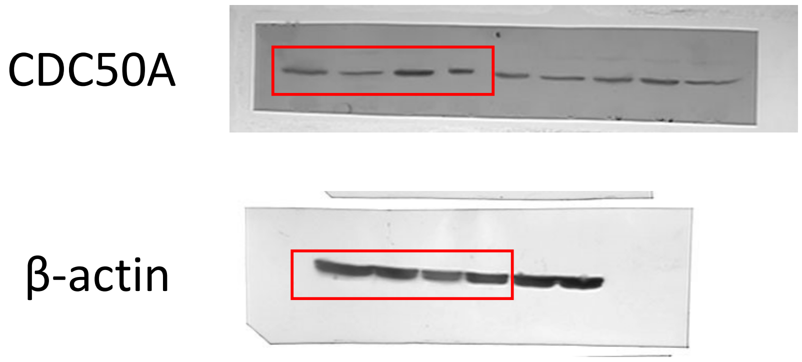

Supplementary Figure 6. The raw data of Figure 2, panel A. The region used in Figure 2 was marked with red box. Chemiluminescence strip was exposed on Kodak film. Then the image was generated by camera. The blots were cut prior to hybridisation with antibodies. The edge of all images have been exhibited.

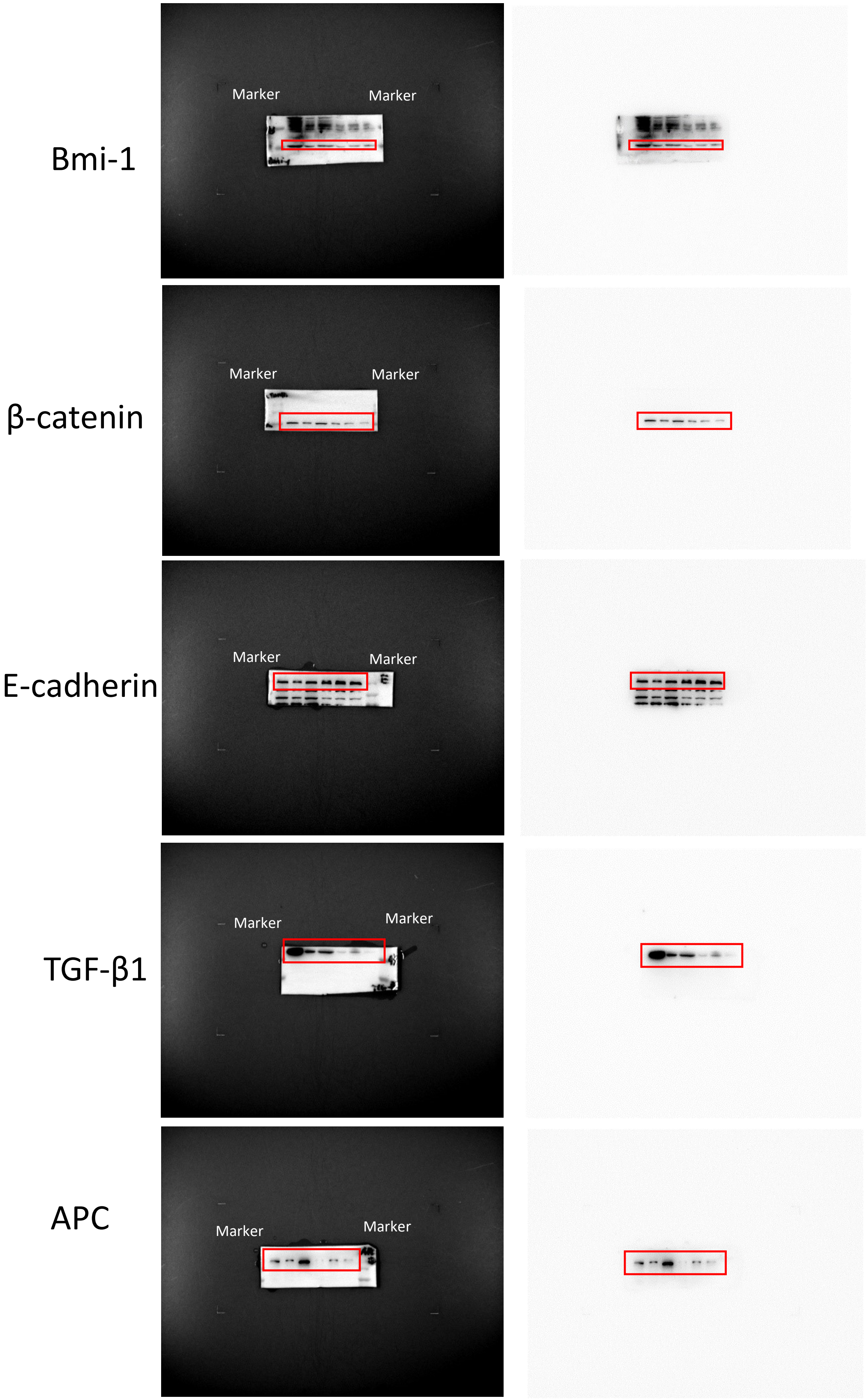

Supplementary Figure 7. The raw data of Figure 3, panel F. The region used in Figure 3F was marked with red box. The left three samples in red box were CDC50A^{+}, the right three were CDC50A^{-}. Marker was sampled on both sides of all samples. Gel was cut before protein transfer membrane. Middle lines of the markers were the both sides of gel. The blots were cut prior to hybridisation with antibodies. The images were generated by chemiluminescence image analysis system (Tanon 520, Shanghai, China). The right was the chemiluminescence exposure image. The left was the merge of daylight image with edge of blots and chemiluminescence exposure image.

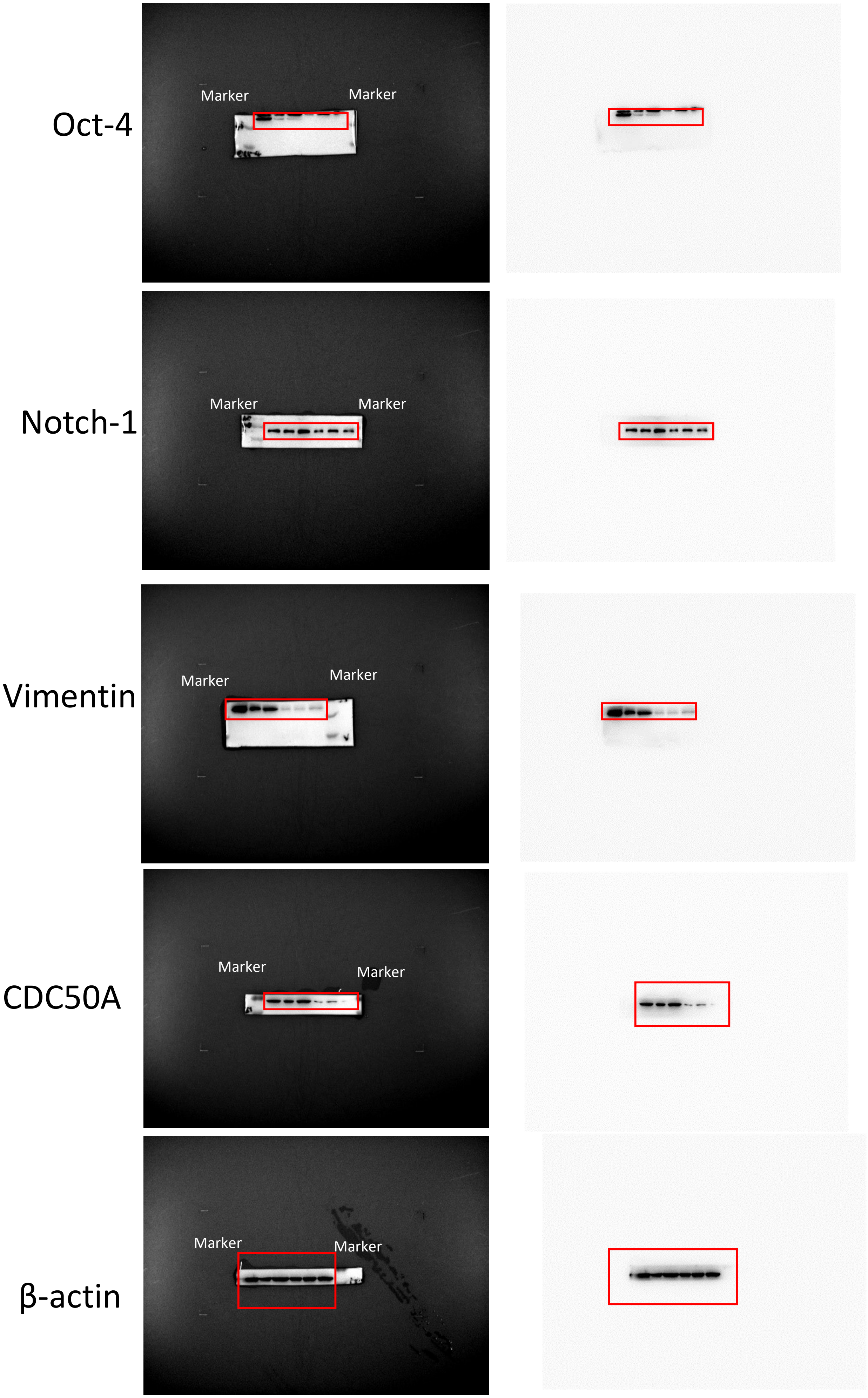

Supplementary Figure 8. The raw data of Figure 3, panel F. The region used in Figure 3F was marked with red box. The left three samples in red box were CDC50A$^+$, the right three were CDC50A$^-$. Marker was sampled on both sides of all samples. Gel was cut before protein transfer membrane. Middle lines of the markers were the both sides of gel. The blots were cut prior to hybridisation with antibodies. The images were generated by chemiluminescence image analysis system (Tanon 520, Shanghai, China). The right was the chemiluminescence exposure image. The left was the merge of daylight image with edge of blots and chemiluminescence exposure image.

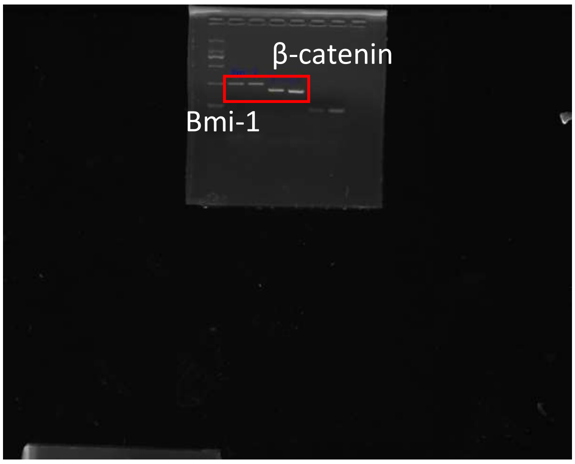

Bmi-1, β-catenin

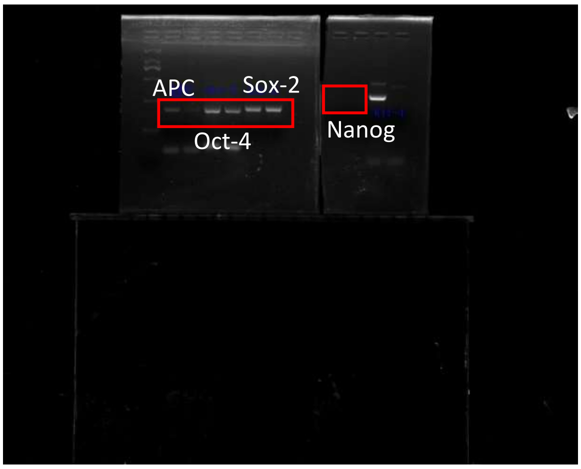

APC, Oct-4, Sox-2, Nanog

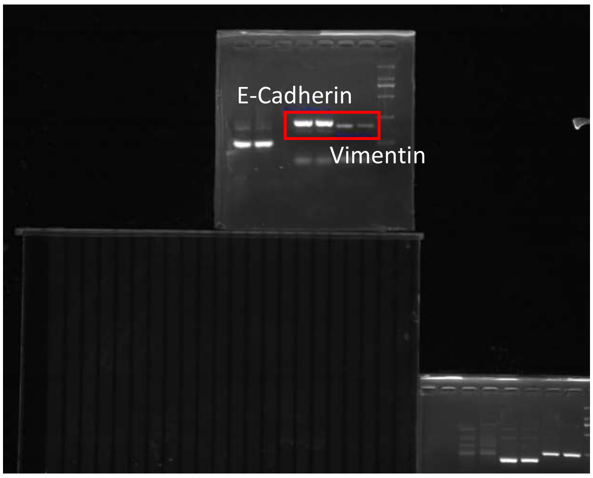

E-Cadherin, Vimentin

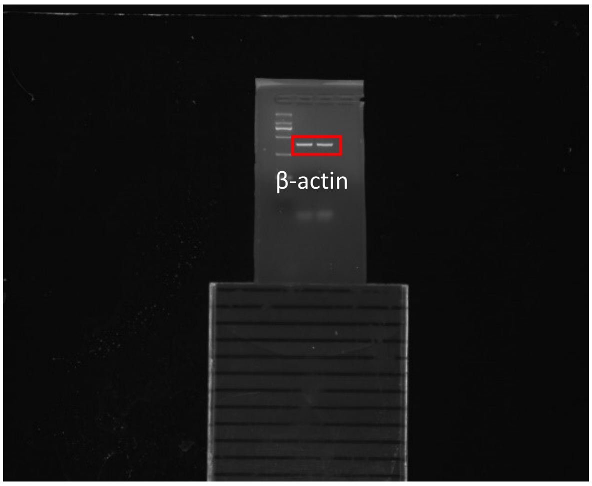

β-actin

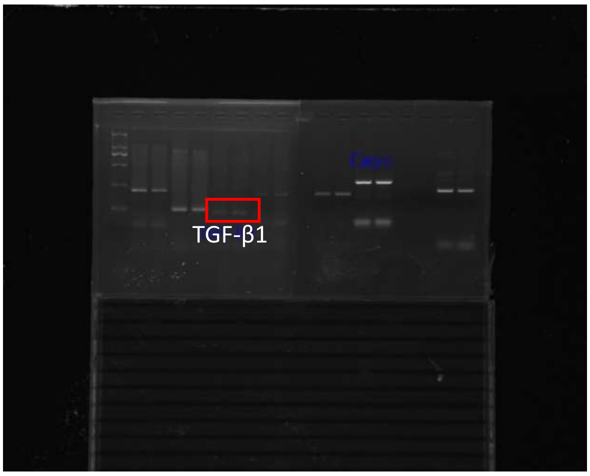

TGF-β1

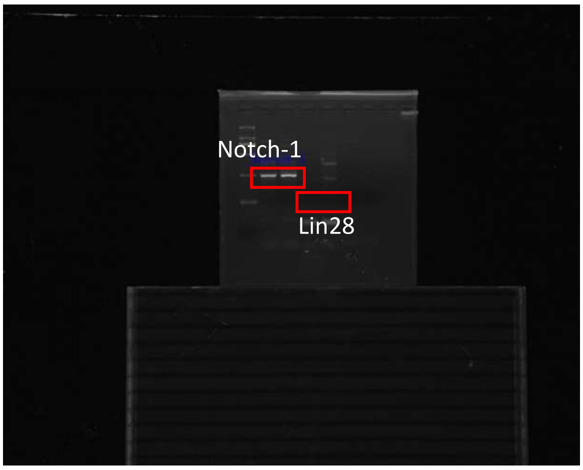

Notch-1

Supplementary Figure 9. The raw data of Figure 4, panel D. The region used in Figure 3F was marked with red box. The left band of Bmi-1, β-catenin, APC, Oct-4, Sox-2, Nanog, E-Cadherin, Vimentin, Notch-1, Lin28 and β-actin were CDC50A^{+}, and the right was CDC50A^{-}. The left band of TGF-β1 was CDC50A^{-} and the right was CDC50A^{+}.

CDC50A

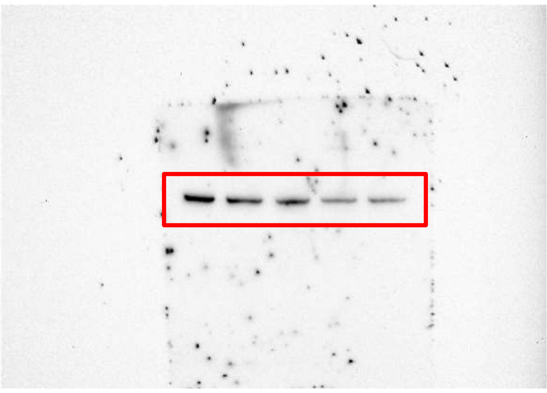

β-actin

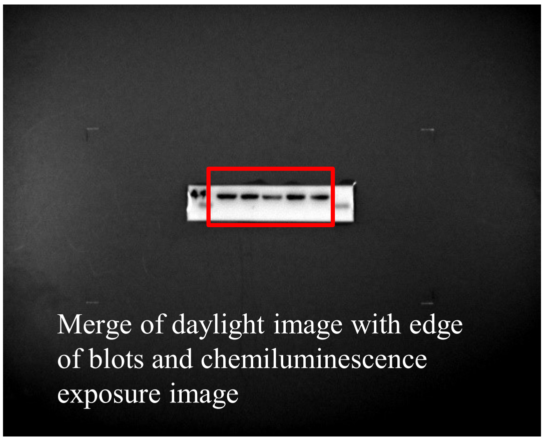

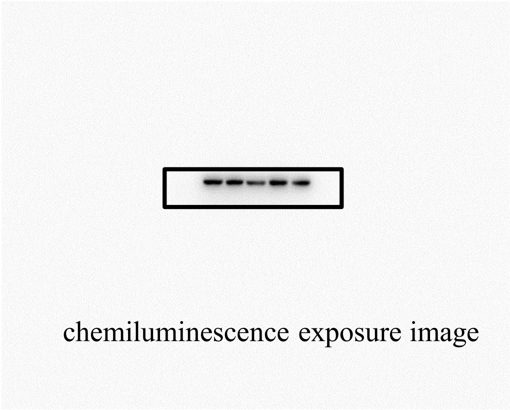

Supplementary Figure 10. The raw data of Supplementary Figure 1. The image is generated by the chemiluminescence imaging system (Tanon 520, Shanghai, China). The blots were cut prior to hybridisation with antibodies. The whole raw images have been exhibited. The region used in Supplementary Figure 1 was marked with red box. The edges of the blots (β-actin) in chemiluminescence exposure image were outlined with solid black lines.

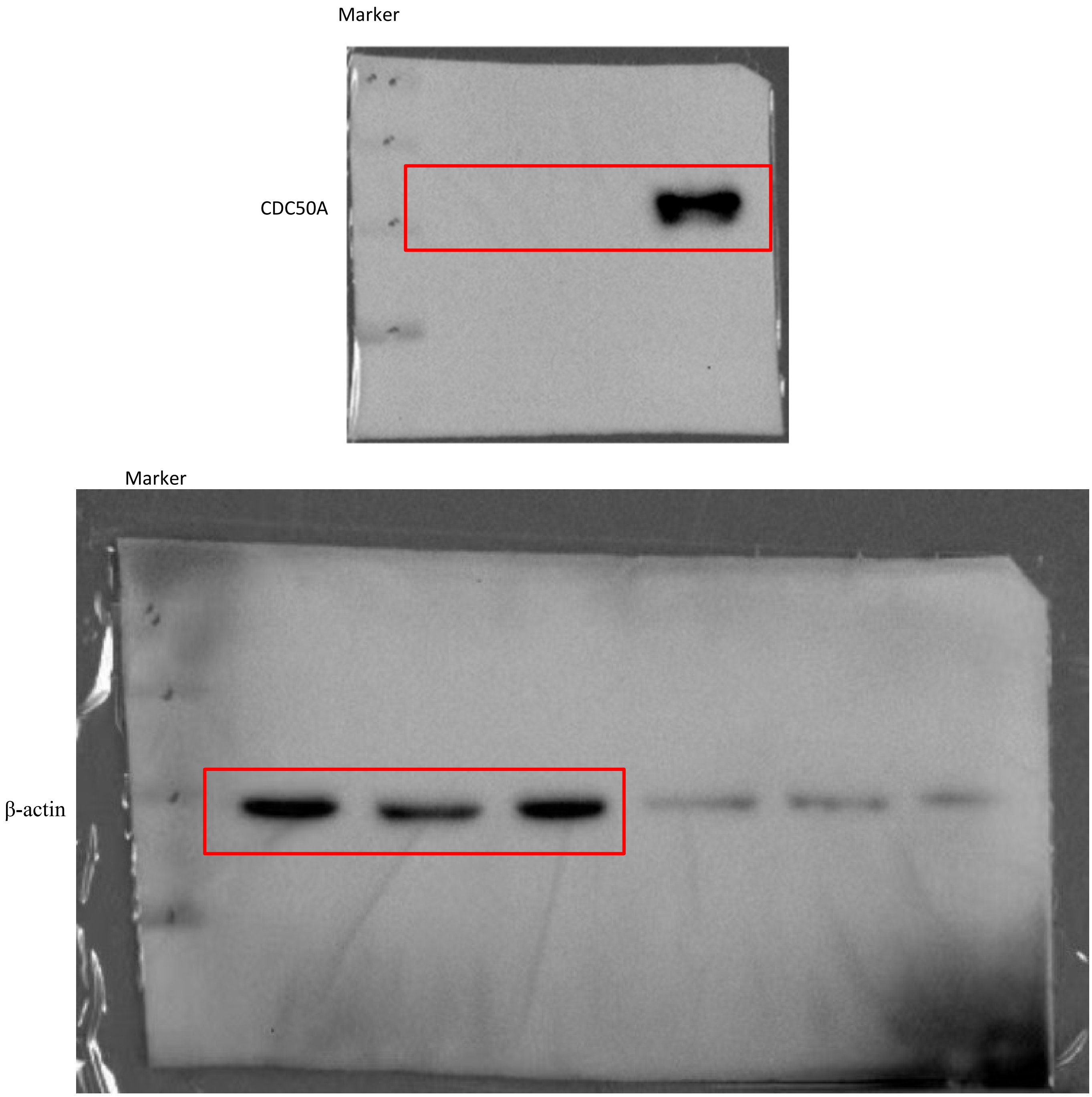

Supplementary Figure 11. The raw data of Supplementary Figure 2, panel B. Chemiluminescence strip was exposed on Kodak film. The image was generated by camera. The blots were cut prior to hybridisation with antibodies. The edge of all images have been exhibited. The region used in Supplementary Figure 2B was marked with red box.
